# Viral Ejection Proteins: Mosaically Conserved, Conformational Gymnasts

**DOI:** 10.3390/microorganisms10030504

**Published:** 2022-02-24

**Authors:** Nicholas A. Swanson, Chun-Feng D. Hou, Gino Cingolani

**Affiliations:** 1 Department of Biochemistry and Molecular Biology, Thomas Jefferson University, 1020 Locust Street, Philadelphia, PA 19107, USA; nswan@mit.edu (N.A.S.); chun-feng.hou@jefferson.edu (C.-F.D.H.); 2 Department of Biology, Massachusetts Institute of Technology, 31 Ames Street, Cambridge, MA 02139, USA

**Keywords:** ejection proteins, DNA ejectosome, viral genome ejection, cell envelope, bacteriophage T7, *Podoviridae*, internal core proteins, gp14, gp15, gp16

## Abstract

Bacterial viruses (or bacteriophages) have developed formidable ways to deliver their genetic information inside bacteria, overcoming the complexity of the bacterial-cell envelope. In short-tailed phages of the *Podoviridae* superfamily, genome ejection is mediated by a set of mysterious internal virion proteins, also called ejection or pilot proteins, which are required for infectivity. The ejection proteins are challenging to study due to their plastic structures and transient assembly and have remained less characterized than classical components such as the phage coat protein or terminase subunit. However, a spate of recent cryo-EM structures has elucidated key features underscoring these proteins’ assembly and conformational gymnastics that accompany their expulsion from the virion head through the portal protein channel into the host. In this review, we will use a phage-T7-centric approach to critically review the status of the literature on ejection proteins, decipher the conformational changes of T7 ejection proteins in the pre- and post-ejection conformation, and predict the conservation of these proteins in other *Podoviridae*. The challenge is to relate the structure of the ejection proteins to the mechanisms of genome ejection, which are exceedingly complex and use the host’s machinery.

## 1. Introduction

Bacteriophages (phages/viruses that infect bacteria) are the most diverse and abundant biological entities on the planet [1]. Outnumbering bacteria in some ecosystems by ten-fold and found in every biome, current estimates point to a staggering 10^31^ phages, which is larger than the currently known number of stars in the universe [2,3]. Phages have evolved several mechanisms to deliver their hydrophilic genomes through hydrophobic membranes and periplasmic environments [4]. About 96% of prokaryote viruses have tails implicated in many viral-life-cycle steps, suggesting an evolutionary advantage in breaking the icosahedral capsid symmetry [5]. Accordingly, phage-tail morphology has inspired a common classification of tailed phages, which are usually divided into three broad morphotypes: *Myoviridae*, characterized by long, contractile tails such as the popular spider-like phage T4; *Siphoviridae,* which have long, non-contractile and floppy tails such as phage lambda; and *Podoviridae*, which contain short, non-contractile tails such as T7 and P22 [6,7]. Phage adsorption on the target cell is mediated by a receptor, which can be proteins or sugars, that position the phage for DNA ejection [8]. Across tailed bacteriophages, interactions with receptors often trigger drastic conformational changes resulting in phage genome ejection [9,10,11].

The mechanisms of DNA ejection are moderately understood and have been reviewed in depth for *Myoviridae* and *Siphoviridae*, whereby the tail undergoes a variety of dramatic conformational changes upon the recognition of a cell-exposed receptor(s) [12,13]. *Myoviridae* use contractile sheaths that puncture the host envelope, allowing the direct transmission of their genomes [14], whereas *Siphoviridae*, filamentous phages, and other tail-less viruses often utilize host membrane channels [15,16,17]. In contrast, *Podoviridae* are less understood, possibly because of their short, non-contractile tails and their lack of a known proteinaceous receptor [11,18,19,20]. *Podoviridae* share the challenge of crossing a host bacterial-cell envelope with tails that can be shorter than the cell envelope itself [21,22]. Furthermore, the periplasmic space of Gram-negative bacteria is a harsh oxidizing environment that contains a peptidoglycan cell wall and houses several host enzymes that are capable of degrading phage DNA [23,24,25,26]. To overcome this physical barrier, *Podoviridae* phages carry their own genome-delivery conduit assembled from a set of internal virion proteins, named ejection or pilot proteins, that are required for infectivity [19]. Ejection proteins are packaged along with double-stranded DNA (dsDNA) in the procapsid, and there is evidence that the intravirion space can be occupied by packaged DNA when they are absent [27]. The position of ejection proteins in the capsid has been a subject of intense investigation. These proteins can be dispersed throughout the capsid [12], exist in ordered structures above the portal protein [28,29,30,31,32] or loosely connected to the protein barrel [33], or even reside inside the tail complex [34]. Upon infection, ejection proteins are expelled inside the host, before the phage DNA [35], where they assemble a transient channel in the host-cell-envelope virion [34,36,37]. Assembling a machine inside the bacterial-cell envelope poses two fundamental challenges.

On the one hand, the ejection proteins must be available for immediate expulsion and coordinated assembly into the host. On the other hand, the genome-delivery conduit must only transiently disrupt the host-cell envelope, allowing the envelope to heal post-genome translocation [18,38,39]. The DNA ejectosome may disassemble once the viral genome has completely entered the host cytoplasm. If the created phage machinery constitutively opens pores, then the host membrane potential and cellular integrity would be disrupted, leading to premature lysis prior to viral replication, sometimes called “lysis from without” [40,41,42,43]. Perhaps due to these challenges, ejection proteins have remained generally understudied compared to the classical structural components of a phage capsid. Here, we review the current understanding of ejection proteins in *Podoviridae* using the prototypical *Escherichia coli* phage T7 as a model system. Additionally, we explore the conformational gymnastics of T7 ejection proteins and their conservation across *Podoviridae*.

## 2. The T7 DNA Ejectosome

**Figure 1:**
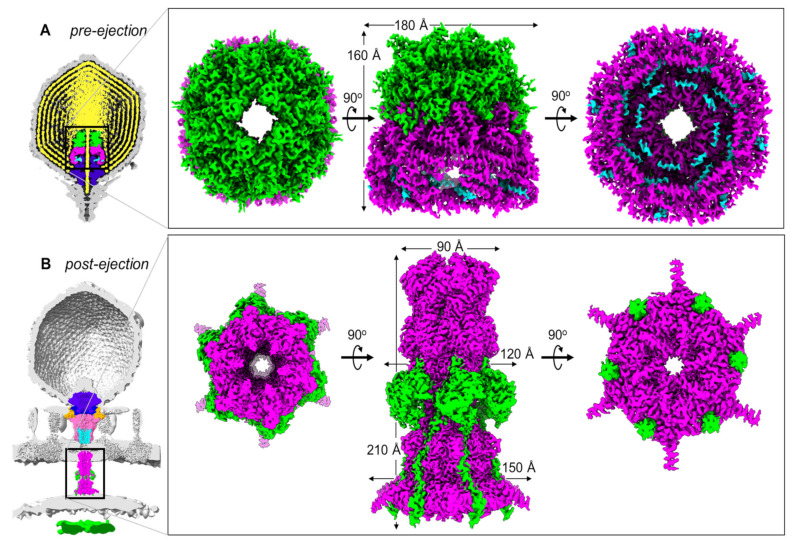
T7 ejection proteins cryo-EM maps. (**A**) Slice through the cryo-EM reconstruction of the T7 mature virion (map EMD-31315) revealing the pre-ejection conformation of gp14 (cyan), gp15 (magenta), and gp16 (lime green) assembled into a core stack (PDB id 7EYB) on the portal protein (purple) and surrounded by the viral genome (yellow). (**B**) Slice through the cryo-EM reconstruction of LPS-treated empty T7 phage (map EMD-31318) superimposed onto the cryo-ET reconstruction of T7 infecting *E. coli* minicells (map EMD-5534). The structure of T7 gp15:gp16-N (PDB id 7K5C) was overlaid with the cryo-ET density. Other proteins: portal (purple; PDB id 7EY6), tail complex (gp17 tail fibers in orange, gp11 adaptor in dark blue, gp12 nozzle in hot pink; PDB id 7EY7), gp14 (cyan; PDB id 7EY7). The zoom-in panel shows the structure of the gp15:gp16-N complex assembled to form a periplasmic tunnel.

The *Escherichia coli* phage T7 contains three internal core proteins, gp14, gp15, and gp16, which are essential for phage morphogenesis and translocation of the ~40 kbp T7 genome [18,19,44,45,46,47]. They account for ~1.5 MDa of mass inside the mature T7 virion (Figure 1A) and, upon infection, are expelled through the narrow portal channel along the portal-tail vertex [36,48,49]. The T7 DNA ejectosome assembles after a virion encounters a host cell that triggers gp14, gp15, and gp16 ejection into the host periplasm [50,51,52]. The tomographic reconstruction of T7 infecting *E. coli* minicells revealed a tube-like density spanning the periplasm that was also accompanied by a toroid density inside the host cytoplasm (Figure 1B) [36]. Like T7, both *Salmonella*-phages P22 [34] and Epsilon 15 [39] eject internal virion proteins inside the host upon infection, resulting in a tail-like apparatus. Additionally, the phage P-SSP7 [53,54] inner core disappears after host attachment concomitantly with the appearance of a tube-like density inside the host-cell envelope.

Recent in vitro studies have characterized the composition of the T7 ejection proteins and elucidated the high-resolution cryo-EM structures of gp15 and an N-terminal portion of gp16 (gp16-N) [55,56], which form a hexameric tunnel wide enough for dsDNA to pass through, bridging the outer membrane (OM) with the inner membrane (IM). In parallel, a recent high-resolution structure of the mature T7 virion [31] elucidated the atomic structures of the T7 ejection proteins in situ, arranged onto the portal protein. It became clear that T7 ejection proteins exist in two structurally distinct states: a pre-ejection conformation, in which they are coaxially arranged as rings on the portal to form a ‘core stack’ [28,29,30,31,32,36], and a post-ejection conformation, assembled as a transenvelope channel in the host-cell envelope called the DNA ejectosome [36,55,56,57].

Below, we will compare and contrast the two pre-/post-ejection states of T7 ejection proteins to decipher how the structures relate to the conformational dynamics and stability of the resulting oligomers.

## 3. Conformational Gymnastics of T7 Ejection Proteins

Gp14 forms a pore in the OM: Gp14, the smallest ejection protein (196 residues), is entirely water-insoluble in vitro, requiring detergents to be extracted from the expression host’s membranes [47,58]. In the pre-ejection conformation, gp14 is solubilized by gp15 as part of the core stack [31] (Figure 1A). The N-terminus of gp14 (residues 1–78) was visualized by cryo-EM inside gp15’s dome-like structure, but due to the peculiar ring-like arrangement of gp14 and gp15 and the eight-fold symmetry used in the reconstruction, it was not possible to conclusively assign a copy number for gp14 that could be present in 8–20 copies [28,29,30]. Biochemical studies confirmed that gp14 is the first internal core protein to be ejected into the host upon virion adsorption on host membranes or rough lipopolysaccharides (LPS) [47]. Its sub-cellular localization post-infection was determined to be the host outer membrane by membrane fractionation [47]. Accordingly, in the cryo-EM reconstruction of the core stack, gp14 is positioned closest to the exit channel, suggesting it may be the first ejection protein expelled into the host. A partial structure of the gp14 post-ejection conformation (residues 37–139) revealed a hexameric helical channel [31]. Accordingly, detergent-solubilized gp14 has pore-forming activity in vitro [55,58]. The limited structural information on gp14 pre- and post-ejection prevent a detailed analysis of the structural changes occurring upon ejection. However, the final stoichiometry of gp14 after ejection is consistent with a hexameric channel [31], which forms a constitutively open channel when inserted into a lipid bilayer [55].

**Figure 2:**
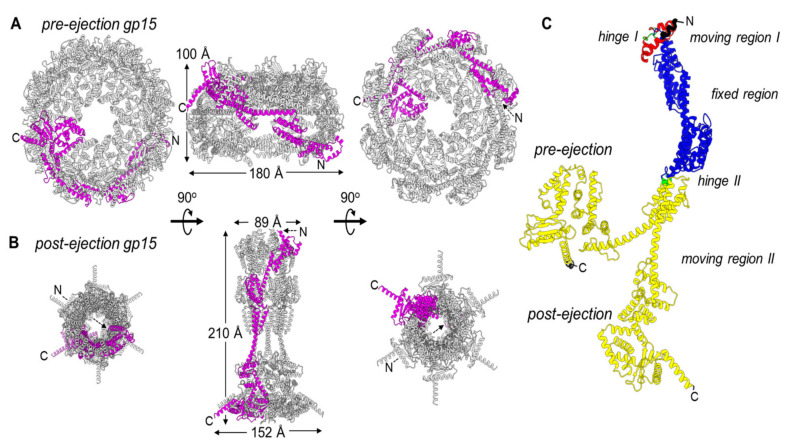
Gp15 conformational gymnastics from pre- to post-ejection. (**A**) Octameric assembly of gp15 in the pre-ejection internal core stack shown in top, side, and bottom views. (**B**) Hexameric gp15 assembly in the post-ejection conformation. In both panels A and B, only one gp15 protomer is colored in magenta. (**C**) DynDom analysis of gp15 flexible regions. Residues colored in blue are superimposable (RMSD ~ 2 Å), red and green move significantly (RMSD > 4 Å), and the entire gp16-C, in yellow, undergoes dramatic restructuring (RMSD > 10 Å). The residues in black, not modeled in the pre-ejection conformation, were not analyzed.

Gp15 straightening: T7 gp15 (747 residues) is mainly monomeric in solution, at low concentration, and displays DNA-binding activity [49,55]. At higher concentrations, gp15 forms an oligomer of ~540 kDa consistent with a hexameric assembly [56]. Spectroscopic studies revealed that gp15 is highly folded and enriched with α-helices but lacks predicted transmembrane helices or liposome-binding activity [50,59]. The complete gp15 atomic structures in the pre- and post-ejection states are available for direct structural comparison. In the pre-ejection conformation [31] (Figure 2A), gp15 forms a dome-shaped assembly of approximately 180 Å (width) by 100 Å (height). The quaternary structure is built by eight arched protomers that adopt a banana-shaped conformation. The globular organization of the gp15 stack is stabilized by extensive lateral contacts (Figure 2A). Upon T7 adsorption on the host, gp15 associates with the cell fraction [47] and has been hypothesized to be a significant constituent for tail lengthening, possibly spanning the length of the periplasm with gp16 [46,52,57]. The post-ejection structure of T7 gp15 bound to gp16-N [55,56] revealed an elongated and slender morphology, with an overall height of ~200 Å, more than twice its width of ~60 Å (Figure 2B). This drastic reorganization results from two coordinated and possibly sequential events: a tertiary-structure rearrangement of the gp15 protomer that changes from an arch to a stick conformation (Figure 2C) that is less than 30 Å in width, possibly narrow enough to fit through the T7 portal protein [48] during ejection; a quaternary-structure change of gp15 from octamer to hexamer, with the loss of two gp15 subunits in the post-ejection state. We will analyze these two events independently.

To determine the regions that undergo the most significant restructuring upon ejection at the tertiary-structure level, we subjected the gp15 protomer structures in pre- and post-ejection conformations to domain-motion analysis using the program DynDom [60] (Figure 2C). We found that gp15 N-termini (residues 95–303) undergo the least motion, with an RMSD of 2.4 Å between the two conformations (colored in blue in Figure 2C). In contrast, the gp15 C-terminal region spanning residues 304–704 is significantly more flexible than the N-terminus with an RMSD of 13.8 Å in the two states. This C-terminal region undergoes a 95.1° angle rotation around a hinge at residues 305–306 (colored in green in Figure 2C) that positions the C-termini ~20.5 Å away from each other. Interestingly, gp16 stabilizes gp15 C-termini in the post-ejection conformation. A second hinge movement occurs at the far N-terminus of gp15 (colored in red in Figure 2C), where residues 67–91 undergo a 4.3 Å translation and a 128.5° rotation in the two states. This region is likely associated with gp14 after ejection into the host.

The restructuring of gp15 protomers occurs concomitantly with ejection, leading to a change in the quaternary-structure stoichiometry from octameric to hexameric. Three lines of evidence corroborate the hexameric oligomeric state as physiological. First, two independent groups reported this stoichiometry using different purification and assembly procedures [55,56,58]. Second, gp15 extends the gp14 pore, which is hexameric after ejection from the virion into the host-cell envelope [61]. Third, the analysis of gp15 thermodynamic stability in the octameric pre-ejection structure versus the hexameric post-ejection conformation suggests the latter has enhanced stability. In the post-ejection conformation, the N-terminal region of gp15 makes five new inter-residue contacts, and the C-terminal region makes eighteen new contacts for a total of twenty-three new contacts formed. The formation of new intra-protomer contacts during post-ejection stabilizes the hexameric quaternary structure that is more thermodynamically stable than the loosely octameric assembly formed in the virion. It is unknown whether the two gp15 subunits that are lost in the post-ejection state are ejected at all from the virion or lost in the host periplasm during assembly of the DNA ejectosome.

**Figure 3:**
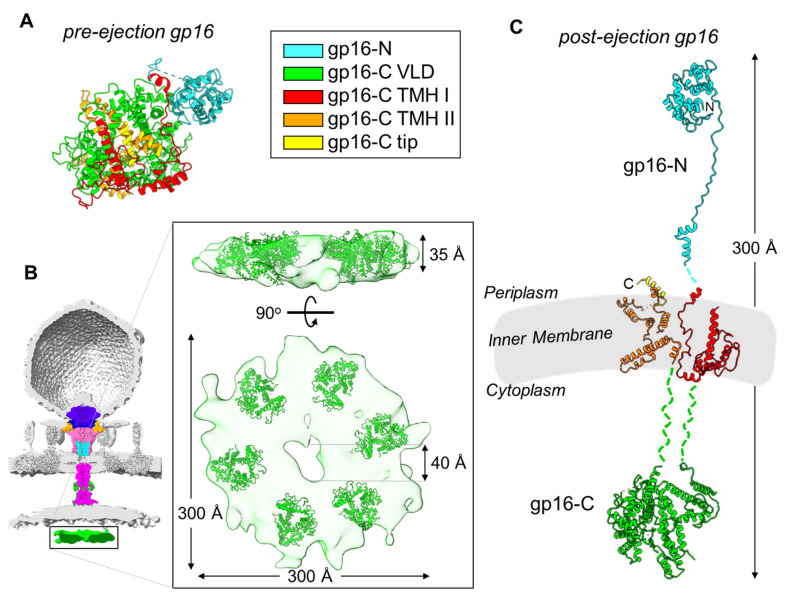
Modelling gp16 post-ejection. (**A**) Tertiary structure of gp16 in the pre-ejection conformation, color coded based on the mapped domains (PDB id 7EYB). (**B**) Six copies of gp16-C (residues 411-1118; PDB id 7EYB) were fit inside the toroid density observed inside the host cytoplasm (colored in green; map EMD-5534). The density was exacted from the cryo-ET reconstruction shown as a semitransparent surface. (**C**) Composite model of gp16 tertiary structure in the post-ejection conformation, colored as in panel A. Only 16-N was experimentally observed. Length of post-ejection conformation gp16 estimated from cryo-ET reconstructions of T7 infecting *E. coli* minicells [36].

Gp16 refolding: T7 gp16 is nearly double the size of gp15 with 1318 residues. It purifies as a soluble monomer [50,55,58] despite having two predicted transmembrane helical regions [50]. Gp16 is largely α-helical in the pre-ejection conformation and can refold from a partially unfolded state as expected for exit from the narrow portal channel [19,47,50]. In the core stack, gp16 is arranged in the top ring, located furthest from the exit channel [28,29,30,36,46], where it adopts a globular structure in the pre-ejection state (Figure 3A). Four subunits related by four-fold rotational symmetry are visible in the procapsid [30] and mature-virion [28,29,31] reconstructions, although more subunits that do not obey strict rotational symmetry could be present inside the head and invisible to cryo-EM analysis. Only the N-terminal portion of gp16, named gp16-N (residues 1-228), was detected at high resolution in the post-ejection conformation (Figure 1B) [55], whereas gp16-C was visualized at low resolution in the lipid nanodiscs [55] and by cryo-ET [36], preventing direct comparison of the pre-ejection state that was visualized in situ in the mature T7 capsid [31].

We used all of the structural and biochemical information available in the literature to generate a composite model of the full-length gp16 in the post-ejection conformation (Figure 3C). To illustrate the rationale behind this modeling, we will divide gp16 into three regions that span the periplasm, IM, and host cytoplasm. First: the gp16 periplasmic portion is known from cryo-EM studies [55,56]. The gp16 N-terminal residues 1–156 form the wings of the gp15 tunnel and contain the transglycosylase domain that is necessary for genome internalization under certain conditions of highly cross-linked peptidoglycan [61,62]. This domain is followed by an extended region (residues 157–228, or ‘molecular tape’) that cements the gp15 binding interfaces and stabilizes the hexameric conformation. Second: the gp16 C-terminal domain is presumed to extend into the host cytoplasm and was assigned to the toroid density visible in the cryo-ET of T7-infected minicells (Figure 3B) [36]. This toroid density, located ~120 Å below the bacterial IM, has approximate dimensions of 300 × 60 Å with a ~40 Å central cavity [36]. We placed six copies of gp16-C (residues 411–1118) in the toroid density, which provides a template by which to define the cytoplasmic topology of this ejection protein (Figure 3B). The location of gp16-C inside the host cytoplasm is supported by a wealth of in vitro and in vivo studies. Gp16-C has sequence-independent DNA-binding activity in vitro [50,55,58]. Missense mutations within a 127-residue stretch of gp16-C such as G737D, I754T, and E761K result in transcription-independent genome translocation of the entire 40 kbp genome, which has led to the hypothesis of a role for gp16-C in clamping the viral genome around 850 bp upon entry into the host [18,45,63,64]. Third, gp16 has two putative transmembrane regions named TMH I (residues 243–410) and THM II (residues 1119–1290) that likely mediate gp16’s ability to insert into liposomes in vitro [36,50], which has also been observed for the gp16 homolog in P22 [65]. Gp16 THMs are globularly folded in the pre-ejection conformation (Figure 3A) but refold in the post-ejection state to cross the inner bacterial membrane twice. It is unknown if these two regions form a complex or remain unassociated inside the host lipid bilayer. Gp16 does not have pore-channel activity in isolation or when bound to gp15 [55,58], suggesting that a channel for DNA passage transiently forms at the IM. In addition, gp16-C is soluble in solution but can also associate with lipid nanodiscs and is visible by cryo-EM [55,58]. The overall length of the post-ejection gp16 is about 300 Å, and the protein remarkably spans the periplasm, the IM, and the host cytoplasm (Figure 3C).

**Figure 4:**
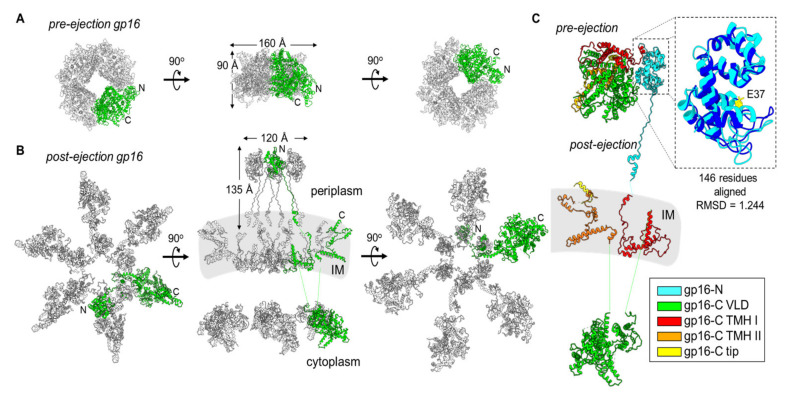
Gp16 conformational gymnastics from pre- to post-ejection. (**A**) Tetrameric assembly of gp16 in the pre-ejection internal core stack shown in top, side, and bottom views. (**B**) Hexameric gp16 assembly in the post-ejection conformation fit within the cryo-ET volume for T7 infecting *E. coli* minicells (middle panel; map EMD-5534). Only 16-N was experimentally observed. Gp16-C and the transmembrane regions were modelled as shown in Figure 3. In both panels A and B, only one gp16 protomer is colored in green. (**C**) Superimposition of pre- and post-ejection conformations of gp16 colored as in Figure 3. Zoom-in panel of residues 11–156 of pre- and post-ejection gp16-N aligned (RMSD 1.244) and colored in cyan and dark blue, respectively. The conserved transglycosylase fold’s catalytic glutamic-acid residue, E37, is highlighted in yellow.

Using the composite model of the gp16 tertiary structure (Figure 3C) and knowing the hexameric quaternary structure of gp16-N in the post-ejection conformation [55] (Figure 1B), we built a quaternary-structure model of the full-length gp16 after genome ejection (Figure 4B,C). This analysis revealed that gp16 undergoes a large refolding upon insertion into the IM, adopting a double-ring-like structure. The refolding liberates the N-terminal domain (gp16-N) that resides in the periplasm, while the C-terminal domain implicated in DNA binding protrudes into the host cytoplasm [36]. Unbundling these two globular domains exposes two putative transmembrane regions (in red and orange in Figure 3C) that may form a channel for genome ejection that is only open during phage infection [55]. Six copies of gp16 are found in the post-ejection conformation, in contrast to the four copies visible in the core stack (Figure 4A). It has been suggested [55] that two additional copies of gp16 present inside the virion but not symmetrically arranged in the core stack are ejected into the host upon infection, analogous to phage P22, where gp16, which is not part of the portal hub, is loosely bound near the portal-protein barrel [33].

## 4. Conservation of Ejection Proteins

Genes encoding ejection proteins are commonly found in *Podoviridae* phages that infect Gram-negative bacteria, including Enterobacteriaceae, Mycobacteria, Pseudomonadaceae, and Cyanobacteria. However, they are not found in phi29-like phages, which are also members of the *Podoviridae* family that infect Gram-positive bacteria and that have a completely different cell envelope consisting of only one lipid membrane and a thicker peptidoglycan layer. Using phage-T7 ejection proteins as a reference, we bioinformatically identified the genes encoding the homologous gp14, gp15, and gp16 proteins in fourteen *Podoviridae* family members that infect *Escherichia coli* (T7, CUS-3, 13a, BA14, K1E, HK620), *Salmonella* (P22, Epsilon15, SP6), *Shigella* (Sf6), *Prochlorococcus* (P-SSP7), *Klebsiella* (K11)*, Yersinia* (phiYeO3-12), and *Pseudomonas* (phiKMV) (Table 1 and Tables S1–S3). The ejection-protein genes are clustered in a small operon, where the gene encoding the gp14-like factor is adjacent to gp15, followed by a larger ORF encoding gp16. We found a marked divergence in the size of gp15 and gp16 versus gp14. Gp15 can vary by as much as 138% among different phages, from 431 aa in Sf6 to 982 aa in phage K1E. Similarly, gp16 varies in size by as much as 120%, from 609 aa in P22 to 1337 aa in phage phiKMV. In contrast, gp14 is more consistent and varies in size by less than 30% (from 181 aa in phiKMV to 240 aa in K1E) (Table 1). In general, P22-like phages (e.g., P22, Sf6, HK620, CUS-3) [66] appear to have a significantly smaller gp15 and gp16 than other *Podoviridae*. This is interesting considering that in P22-like phages, the ejection proteins do not form a core stack in the pre-ejection conformation [34] but are dispersed inside the virion, possibly residing in the proximity of the portal protein [33].

**Figure 5:**
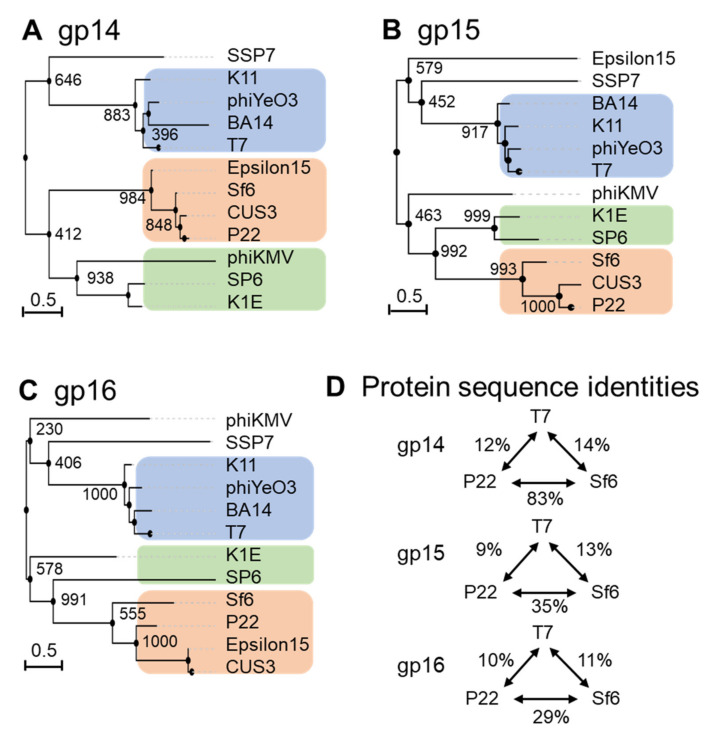
Diversity of the ejection proteins across fourteen representative *Podoviridae*. Phylogenetic trees for (**A**) gp14-like, (**B**) gp15-like and **(C**) gp16-like ejection proteins created using FastME 2.0 and PhyML 3.0 [68,69] with branch support of 1000 bootstrap repeats. (**D**) Table data on sequence identities and similarities with respect to the T7, P22 or SP6 ejection proteins were calculated using SIAS (http://imed.med.ucm.es/Tools/sias.html; accessed on 5 January 2022).

Ejection-protein sequences were aligned with ClustalW [67] and converted to phylip format [66] for phylogenetic-tree calculation using PhyML 3.0 [68]. This analysis revealed that each ejection protein falls into at least three major groups, which have diverged greatly throughout evolution, in a neighbor-joining tree highlighted with different-colored boxes in Figure 5A–C. For ease of description, the three major groups are named by three representative phages: T7, P22, and SP6. Interestingly, the three ejection proteins are the most diverse between P22- and T7-like phages (e.g., T7, 13a, BA14, phiYe03, K11), whereas the SP6 group was clustered with T7 phages for gp16 and P22 phages for gp14 and gp15 (Figure 5A–C). However, Epsilon15 is an out-group in the gp15 tree but clusters with P22 in the gp14 and gp16 trees. This phage has a ‘small’ gp15 and gp16 (Table 1), similar to P22-like phages, but also displays a core stack in the pre-ejection conformation, similar to phage T7 [39]. Below, we will briefly discuss the conservation of each ejection protein.

Gp14 is the most conserved of the three ejection proteins, both in sequence and size (about 200 aa), with an average sequence identity and similarity of 19/29% among the phages analyzed in this study (Figure 5A). T7 phages appear to be more divergent than P22 and SP6 phages, sharing an average sequence identity of 12% and 8%, respectively (Figure 5D). All T7 gp14-like factors contain a set of four or five predicted transmembrane helices, except P-SSP7 gp14, which is predicted to have only two TMHs [55]. These predicted transmembrane α-helices likely allow all gp14 homologs to insert into the host OM, as also revealed by membrane-localization studies in T7 [46,47,49].

Gp15-like homologs vary significantly in size (between 431–982) (Table 1), with an overall sequence identity between 7% and 9% in the three groups identified in Figure 5B. Homology matching to T7 gp15 is challenging as most are matched based on size and synteny rather than demonstrating sequence similarity (Table 1 and Figure 5B) [70]. One study suggested rapid evolutionary divergence occurs for gp15-like proteins as their sequences have diverged to only share 34% identity across 0.4 billion years, whereas on the same time scale, portal protein homologs have retained 69% sequence identity (Figure 5B) [70,71]. Gp15-like homologs are better conserved in close T7 relatives, which have an average sequence identity and similarity of 19/39% (Table 1 and Figure 5B) and even more in P22 phages, with an average sequence identity and similarity of 36/55% (Figure 5B). *Salmonella*-phage P22 gp15-homolog gp20 (471 aa) is ejected upon adsorption on the host and extends the extra-cellular channel across the OM and into the periplasm (Table 1) [34]. Low-resolution structural data and biochemical evidence suggest that P22 gp20 forms a channel extending the tail complex while other ejection proteins span the envelope [34,65,72]. P22 gp20 may also require cleavage by a host enzyme before becoming functional, suggesting that it may be an exception to other homologs (Table 1 and Figure 5B) [65]. Purified *Shigella*-phage Sf6 gp12 (431 aa), which is more closely related to P22 gp20 than T7 gp15, forms a tube-like structure [73] (Figure 5B). Overall, it is likely that all gp15-like homologs are functionally similar, forming the same core tunnel based on consistent secondary-structural elements with some gaps and insertions among close T7 relatives. Surprisingly, some gp15-like homologs such as *E. coli* phages K1E [74] and K1-5 homolog gp35 contain lysozyme activity, which is found on gp16 in the T7 system (see next section) (Table 1 and Figure 5B) [62,74]. The swapping of functional domains across ejection proteins is thematic for the pervasive mosaicism among related phages [72].

Gp16-like homologs also vary significantly in size (between 609–1337) and domain composition [70] (Table 1). T7-like gp16 homologs have an average sequence identity and similarity of 20/40%, which drops to 16/37% among P22-related phages, suggesting rapid divergence between and within these phage groups (Table 1 and Figure 5C). Interestingly, the T7 gp16 N-terminal peptidoglycan-hydrolase domain is not conserved in P22 or Sf6, where this ejection protein is significantly smaller (e.g., 1318 vs. 609 and 665, respectively, Table 1). However, the T7 gp16 putative transmembrane helices at the C-terminus [19,50] were identified in all gp16-like homologs presented in Table 1. Additionally, gp16’s positively charged five C-terminal residues that are necessary for infectivity are also conserved among T7’s closest relatives (Table 1 and Figure 5C) [47]. *Salmonella*-phages P22 and Epsilon15 gp16-like homologs show little sequence similarity to the T7 counterpart despite having matching synteny and implications in forming a tube for genome ejection (Table 1 and Figure 5C) [70,75]. The divergence of gp16 homologs in T7- and P22-like phages suggests the plastic evolution of this protein to solve the challenges of genome delivery [70]. Interestingly, a smaller gp16-C (as in P22-like phages) correlates with the loss of a core stack in the pre-ejection conformation. We speculate that a small C-terminal domain reduces the stability of the gp16 tetramer in the head and its ability to form a defined quaternary structure. Interestingly, the existence of smaller gp15 and gp16 ejection proteins, which are not part of a core stack, as in P22-like phages, correlates with the existence of a portal-protein barrel [76,77]. Intriguingly, in all phages where the ejection proteins are organized into a core stack prior to ejection, the portal protein lacks a C-terminal barrel.

## 5. Models for Ejection-Protein Assembly into a DNA Ejectosome

The exact mechanisms by which T7 expels the ejection proteins and their assembly into a DNA ejectosome remains unknown. Here, we will try to conceptualize recent structures of T7 ejection proteins in pre- and post-ejection conformations and previous biochemical data into two models that we named the “Inverted-Sock model” and the “Octopus model”.

**Figure 6:**
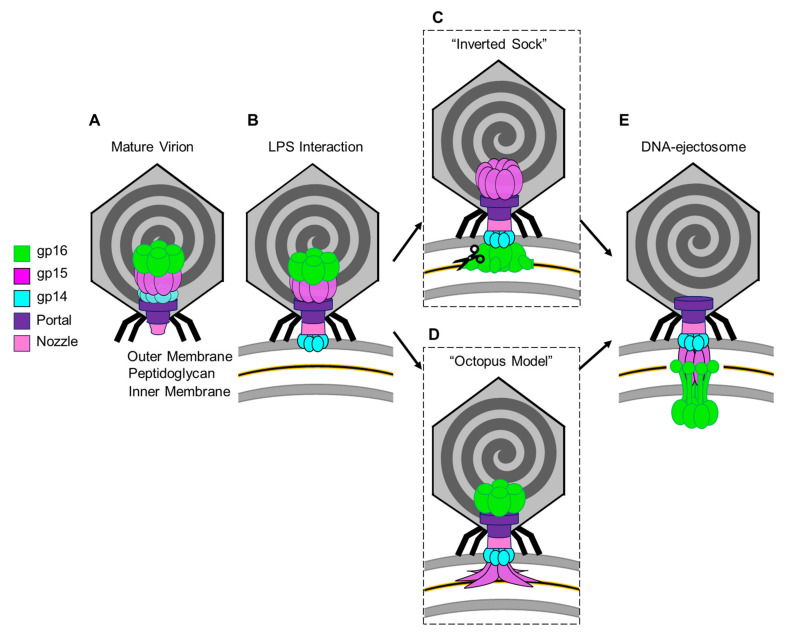
Models for the T7 ejection proteins assembly to form a DNA ejectosome. (**A**)The mature T7 virion shown with the internal core stack concentrically arranged on the portal protein. (**B**) Tail fiber interactions with LPS on the host *E. coli* surface initiate conformational changes in the nozzle and portal protein and eject gp14 to form an outer membrane pore. (**C**) In the “inverted sock” model, gp16 exits next, in the “inverted sock model”, and cleaves the peptidoglycan layer within the host periplasm, followed by gp15’s exit to form the periplasmic tunnel (PT). (**D**) Alternatively, in the “octopus model” gp15 exits after gp14, forming a partially stabilized hexamer prior to gp16’s exit and cleavage of the peptidoglycan barrier in the periplasm. (**E**) Both models end with the formation of the DNA-ejectosome which includes the gp14 outer membrane pore connected to the gp15:gp16 periplamic tunnel which traverses the host inner membrane and projects the gp16-C cytoplasmic hub for viral genome translocation.

The two models differ in the way the ejection proteins assemble into a transmembrane-envelope channel and are based on three assumptions. (i) In the pre-ejection conformation, the internal core is aligned on top of the portal/tail axis (Figure 6A) [31]. (ii) T7 tail-fiber interactions with the host LPS triggers conformational changes within the T7 tail, widening the nozzle to 30 Å, and signals the expulsion of ejection proteins gp14, gp15, and gp16 and the viral genome (Figure 6B) [52]. (iii) The first ejection protein to exit through the portal channel is gp14, which extends the nozzle and inserts into the OM, creating a hexameric, constitutively open pore (Figure 6B) [31,47]. In the “Inverted-Sock model,” we hypothesize that gp16 exits through the portal-tail-gp14 complex channel as a monomer and is ejected into the periplasmic space where it cleaves peptidoglycan via its N-terminal peptidoglycan-hydrolytic domain (Figure 6C). Next, gp15 is expelled as a monomer into the periplasm where interactions with gp14 stabilize its flexible N-terminal domain [56] and interactions with the gp16-N molecular tape stabilize gp15’s flexible C-terminal domain resulting in the gp15:gp16-N hexameric periplasmic tunnel [55]. Expectedly, gp16-C inserts into the host IM, creating a transient pore and projecting a large gp16-C cytoplasmic hub for viral-genome translocation. The “Inverted-Sock model” is aptly named as the transition of ejection proteins from the core stack to the cell envelope mimics the movement of reaching into a sock and inverting it. The “foot” of the metaphorical sock (gp16), which is furthest away from the opening (portal-gp14 OM pore), is pulled through prior to the “tube” of the sock (gp15 forming the periplasmic tunnel tube). In the “Octopus model,” following gp14’s ejection and formation of the OM pore [31], we hypothesize that gp15 exits by forming a stable N-terminal hexameric complex between gp14 in the OM pore and the peptidoglycan barrier with disordered C-terminal arms splayed like an octopus (Figure 6D). Via the portal-tail-gp14-gp15-N connected tunnel, we presume gp16-N to egress and cleave through the peptidoglycan barrier with its transglycosylase activity. Thereafter, gp16-N molecular-tape residues stitch together the disordered gp15 C-terminal regions, assembling the hexameric periplasmic tunnel, which spans the entire periplasm. Gp16-C then breaches the host IM and projects the cytoplasmic hub for viral-genome translocation (Figure 6E).

Both of these intermediary models end with gp16-C ratcheting in the viral genome in a transcription-independent enzymatic manner until the *E. coli* RNApol binds to promoters in the viral genome and initiates translocation via the force of transcription in an energy-dependent manner. The leading stretch of the viral genome that the *E. coli* RNApol transcribes includes the T7 RNApol, which, once assembled, can transcribe the remaining viral genome until completion [19,63]. We expect that once the viral genome has completely entered the host cytoplasm, the DNA ejectosome may disassemble and remove the IM pore formed by gp16-C in order to maintain cytoplasmic membrane potential.

## 6. Conclusive Remarks

In this review, we have taken an inventory of the literature on ejection proteins from the model system of T7 and similar phages. Using a comparative structural analysis of recent cryo-EM snapshots of the T7 ejection proteins, we identified a set of principles that accompany the ejection and assembly of T7 ejection proteins and may serve as a foundation on which to study ejection proteins in other phages:

(i) Gp14 is the first factor to be ejected into the host, where it folds into a hexameric, constitutively open channel embedded in the host OM.
(ii) Gp15 forms the periplasmic tunnel that extends the phage tail to cross the periplasm. The protein undergoes dramatic tertiary- and quaternary-structure conformational changes upon ejection, characterized by straightening of the C-terminal domain that swings by ~128° and assembles into a hexameric DNA tunnel wide enough to accommodate hydrated DNA.
(iii) Gp16, the most complex of the three ejection proteins, has two functions: transglycosylase activity (gp16-N) and cytoplasmic DNA-binding activity (gp16-C). The former is phage-specific (not present in P22-like phages), whereas the latter is universally conserved. Gp16 refolds upon ejection, unbundles, and inserts into the IM to form a dual-ring structure. One ring containing gp16-N is part of the periplasmic tunnel with gp15, while the second ring projects into the host cytoplasm, is active in DNA binding, and takes part in DNA ejection.
(iv) The stoichiometry of the assembly changes upon ejection, with the loss of at least two subunits of gp14 and gp15, which are octameric (or larger) during pre-ejection and become hexameric in the post-ejection state. It is unclear if the additional subunits in the pre-ejection conformation are not ejected from the virion or lost in the periplasm. The gp16 post-ejection conformation is also hexameric, implying additional copies of this protein must exist in the virion but are not visible in the core stack due to the limited volume available in the portal, which accommodates only four copies. These additional copies are likely loosely bound to the portal, as in P22-like phages.
(v) Ejection-protein genes tend to be more variable than other virion-assembly proteins with conservations of under 10% in protein sequences, even in phages that infect the same bacterium. There does not appear to be conservation based on hosts, and ejection proteins that cluster into a core stack in the pre-ejection conformation are not necessarily more similar to one another than those diffused inside the capsid, as in P22-like phages.
(vi) Membrane-spanning secondary-structure elements are universally conserved in gp14 and gp16 homologs, suggesting these two ejection proteins provide anchoring and penetrate the host OM and IM, respectively.
(vii) The N-terminal peptidoglycan-hydrolase domain of T7 gp16 can swap to the gp15 homolog, suggesting a mosaically modular organization and an evolution of ejection proteins whereby the individual components may diverge as long as all parts are present in the final molecular machine.

In conclusion, we are beginning to appreciate the complexity of a new nanomachine, the DNA ejectosome, which is likely much more complex than rationalized in this review. Nonetheless, this review provides a framework for future studies.

## Figures and Tables

**Table 1:** List of gene factors homologous to T7 gp14, gp15 and gp16. Highlighted in bold are the largest and smallest representatives of each group. Annotations for gp15-like and gp16-like gene factors ranging from extra-small (XS) to extra-large (XL) included for comparison within each grouping.

Phage (Host)	gp14-Like (OM)	gp15-Like (Tunnel)	gp16-Like (IM)
T7 (*Escherichia coli*)	gp14 (196 aa)	gp15 (747 aa) M	gp16 (1318 aa) XL
CUS-3 (*Escherichia coli*)	gp7 (230 aa)	gp20 (449 aa) S	gp16 (719 aa) S
13a (*Escherichia coli*)	gp14 (196 aa)	gp15 (747 aa) M	gp16 (1318 aa) XL
BA14 (*Escherichia coli*)	gp14 (201 aa)	gp15 (759 aa) M	gp16 (1315 aa) XL
K1E (*Escherichia coli*)	gp34 (**240** aa)	gp35 (**982** aa) XL	gp36 (1102 aa) M
HK620 (*Escherichia coli*)	gp7 (230 aa)	gp20 (449 aa) S	gp16 (722 aa) S
P22 (*Salmonella enterica*)	gp7 (229 aa)	gp20 (471 aa) S	gp16 (**609** aa) XS
Epsilon 15 (*Salmonella enterica*)	gp11 (229 aa)	gp12 (499 aa) S	gp13 (708 aa) S
SP6 (*Salmonella enterica*)	gp35 (239 aa)	gp36 (978 aa) XL	gp37 (1270 aa) L
Sf6 (*Shigella flexneri*)	gp11 (230 aa)	gp12 (**431** aa) S	gp13 (665 aa) XS
P-SSP7 (*Prochlorococcus marinus*)	gp14 (200 aa)	gp15 (837 aa) L	gp16 (1245 aa) L
K11 (*Klebsiella pneumoniae*)	gp14 (196 aa)	gp15 (751 aa) M	gp16 (1321 aa) XL
phiYeO3-12 (*Yersinia enterocolitica*)	gp14 (197 aa)	gp15 (747 aa) M	gp16 (1320 aa) XL
phiKMV (*Psudomonas aeruginosa*)	gp35 (**181** aa)	gp36 (898 aa) L	gp37 (**1337** aa) XL

## Supplementary Materials

The following are available online at https://www.mdpi.com/article/10.3390/microorganisms10030504/s1, Table S1: Gp14-like sequence alignment, Table S2: Gp15-like sequence alignment, and Table S3: Gp16-like sequence alignment.
