# Supplementary material for: Viral Ejection Proteins: Mosaically Conserved, Conformational Gymnasts
Source: Microorganisms. 2022 Feb 24;10(3):504. doi: 10.3390/microorganisms10030504 (PMC8954989; doi:10.3390/microorganisms10030504)
Supplement: Supplementary file 1 [file microorganisms-10-00504-s001.zip › microorganisms-1605105-supplementary.pdf]

microorganisms

*Supplementary materials*

**Table S1.** Gp14-like sequence alignment.

```
Prochlorococcus_phage_P-SSP7_gp14      -------------------------------------------------------MCLGA        5
Pseudomonas_phage_phiKMV_gp35          -----------------------------------------MAFW--------LPLLAAG        11
Escherichia_phage_K1E_gp34             MAIGSAVMAGMSSIGSMFAGSGAAAAAGGAAAGGGGLLGSLGGFLSGSTAGFSNAGLLGA        60
Salmonella_phage_SP6_gp35              MAIGTALTAGLSSVAGSAASGGFLSS-LGGAIGAEGVMGSAMSFLGGTTGGFSNAGLLSA        59
Salmonella_phage_Epsilon_15_gp11       ---------------------------------------------------MLYAFKLGR        9
Shigella_phage_Sf6_gp11                ---------------------------------------------------MLYAFKLGR        9
Escherichia_phage_CUS-3_gp7-like       ---------------------------------------------------MLYAFTLGR        9
Salmonella_phage_P22_gp7               ---------------------------------------------------MLYAFTLGR        9
Salmonella_phage_HK620_gp7             ---------------------------------------------------MLYAFKLGR        9
Escherichia_phage_BA14_gp14            ---------------------------------------------------MCWMAAIPI        9
Klebsiella_phage_K11_gp14              ---------------------------------------------------MCWMAAIPI        9
Yersenia_phage_phiYeO3-12_gp14         ---------------------------------------------------MCWMAAIPI        9
Escherichia_phage_T7_gp14              ---------------------------------------------------MCWAAAIPI        9
Escherichia_phage_13a_gp14             ---------------------------------------------------MCWSAAIPI        9


Prochlorococcus_phage_P-SSP7_gp14      AA--------K---AA------NENARRRYKYENERRERNWMQTMSIYNAQKV---KYDE        45
Pseudomonas_phage_phiKMV_gp35          GMSA---LQQG---LA------NKEERNKIKAEN---KARLKTDLDNLGAA------A-R        49
Escherichia_phage_K1E_gp34             GLQGLGLIGDL---FG------GSDEAKAMKKAQ---EEQWRQQLIATQEA------Y-K        101
Salmonella_phage_SP6_gp35              GMQMLNPIGDY---FT------QKETAKAMKKAQ---DEQWRQQLIATREA------Y-A        100
Salmonella_phage_Epsilon_15_gp11       KLRGEEPYCAEKGGKGGSSSSGAKEAARATQYAADLQNQQFNRVMEQLAPYAAAGLPALQ        69
Shigella_phage_Sf6_gp11                KLRGEEPYCPEKGGKGGSSDKSAKYAAEAQKYAADLQNQQWQTIMKNLAPFTPLAEQYVN        69
Escherichia_phage_CUS-3_gp7-like       KLRGEEPYYPEKGGKGGSSDKSAKYAAEAQKYAADLQNQQFNTIMNNLKLFTPLAGKYVG        69
Salmonella_phage_P22_gp7               KLRGEEPSYPEKGGKGGA-DKSAKYAAEAQKYAADLQNQQFNTIMNNLKPFTPLADKYIG        68
Salmonella_phage_HK620_gp7             KLRGEEPWCPEKGGKGGSSDKSAKYAAEAQKYAADLQNQQFNTIMNNLKPFTPLADKYVG        69
Escherichia_phage_BA14_gp14            AMQGASMIM---GGMQGEQ------AKAAQIDQGRRQSMQMLKEMNYNE--------ANL        52
Klebsiella_phage_K11_gp14              AMTAVQAIG---QSRNEAK------MIGLQNDQMRRQSAQMIKESNIQN--------ANA        52
Yersenia_phage_phiYeO3-12_gp14         AMMGAQALS---SQNSADK------ARVAQTEAGRRQAMEMVKEMNIQN--------ANA        52
Escherichia_phage_T7_gp14              AISGAQAIS---GQNAQAK------MIAAQTAGRRQAMEIMRQTNIQN--------ADL        52
Escherichia_phage_13a_gp14             ALAGAQAIS---GQNTQAK------MIAAQTAGRRQAMEIMRQTNIQN--------ADL        52
                                                                              .

Prochlorococcus_phage_P-SSP7_gp14      DVQNAGLAQAQVKTDQQEAMDLARGEAQIKY-AELFRK-----------------LLNDS        87
Pseudomonas_phage_phiKMV_gp35          DIANLG-VMA----------ASYRKQAVASQ-VEAKRQ----------G------MLAGG        81
Escherichia_phage_K1E_gp34             TVADAERSAA----------KQYHADAISNQ-ASLLQ--------------------QRA        130
Salmonella_phage_SP6_gp35              SVANAERSAS----------KQYHSELIDNQ-VSLLQ--------------------QRA        129
Salmonella_phage_Epsilon_15_gp11       QIQQLSTL-----EGQNSALNQYYNSEQYKQLADQARYQSLNAAEATGGLGSTATSNQIA        124
Shigella_phage_Sf6_gp11                QLQNLSSL-----EGQGQALNQYYNSQQYKDLAGQARYQSLAAAEATGGLGSTATSNQLA        124
Escherichia_phage_CUS-3_gp7-like       SLENLSSL-----EGQGQALNQYYNSQQYKDLAGQARYQSLAAAEATGGLGSTATSNQLA        124
Salmonella_phage_P22_gp7               SLEGLSSL-----EGQGQALNNYYNSQQYQDLAGQARYQNLAAAEATGGLGSTATSNQLS        123
Salmonella_phage_HK620_gp7             SLENLSSL-----EGQGQALNQYYNSQQYKDLAGQARYQSLAAAEATGGLGSTATSNQLA        124
Escherichia_phage_BA14_gp14            KLESRDLI-----DSTVQEMTQ----------------------------SNMNRVRNMG        79
Klebsiella_phage_K11_gp14              SLEQKQKL-----EEASSDLTA----------------------------KNLDKVQAMG        79
Yersenia_phage_phiYeO3-12_gp14         SLEQRDAL-----EAASSELTT----------------------------RNMQKVQAMG        79
Escherichia_phage_T7_gp14              SLQARSKL-----EEASAELTS----------------------------QNMQKVQAIG        79
Escherichia_phage_13a_gp14             SLQARSKL-----EEASAELTS----------------------------QNMQKVQAMG        79
                                        :                                                         .

Prochlorococcus_phage_P-SSP7_gp14      TYG-KLVASGQTGQSTRR--RATMDYAKYGRDVSDIARRLTLNDR-ELA-------RKSS        136
Pseudomonas_phage_phiKMV_gp35          SAEAQAGAFGVKGASVDA--VALDIEREVGEALIQIDDNLDNQMW-NLAEQAHSIQAQAK        138
Escherichia_phage_K1E_gp34             QVALLAGATGTGGNSVSS--MLNDLAADGGRNQSTIIDNYENQKI-NFTNQLKSIQRGGQ        187
Salmonella_phage_SP6_gp35              QVALLAGASGTGGNSITS--MLNDLTGEAGRNQATIIDNYETQQI-NFANQLKSIQKGGQ        186
Salmonella_phage_Epsilon_15_gp11       SIAPTLGQNWLSGQMQNYGNLLNVGQSAAAGQAS-AGQNYANN------------AGNLA        171
Shigella_phage_Sf6_gp11                TIAPTLGQSWLSNQMSNYNNLANVGLGALQGQAN-AGQTYANN------------MSSIA        171
Escherichia_phage_CUS-3_gp7-like       TIAPTLGQQWLSGQMNNYNNLANIGLGALQGQAN-AGQTYANN------------MSQIS        171
Salmonella_phage_P22_gp7               AIAPTLGQQWLSGQMNNYQNLANIGLGALQGQAN-AGQTYANN------------MSQIS        170
Salmonella_phage_HK620_gp7             TIAPTLGQQWLSGQMNNYQNLANIGLGALQGQAN-AGQTYANN------------MSQIS        171
Escherichia_phage_BA14_gp14            TIRAAIGEGMLEGNSMER--VTRVTEGDFLRESQGLTENYQRDYSVILGKRLANHENTVS        137
Klebsiella_phage_K11_gp14              TIRAAIGEGNLEGASMDR--ISRIEEGKFIREANAVTDNYRRDYASLFAQQLGNSESTID        137
Yersenia_phage_phiYeO3-12_gp14         TIRAAIGEGMLEGESMKR--IKRIEEGNYIREANSVTENYRRDYASIFAQQLGRTQSTAS        137
Escherichia_phage_T7_gp14              SIRAAIGESMLEGSSMDR--IKRVTEGQFIREANMVTENYRRDYQAIFAQQLGGTQSAAS        137
Escherichia_phage_13a_gp14             SIRAAIGESMLEGSSMDR--IKRVTEGQFIREANMVTENYRRDYQAIFAQQLGGTQSAAS        137
                                                   .                             :

Prochlorococcus_phage_P-SSP7_gp14      ---EQISKYKQFKDEAFAKVAFQPIPDVAPPQPVMRNVGAEAFMGALSIASNVATMGGQS        193
Pseudomonas_phage_phiKMV_gp35          ---AGLLGQKSTTAGQ-----RSPLV--------------AGLMSAGSLYASQYFKFGAT        176
Escherichia_phage_K1E_gp34             ---MQMREFK--KPSA-----MSTLV--------------QGIPSLASAYVTGSKSGKAL        223
Salmonella_phage_SP6_gp35              ---MMMRSFE--KPSA-----FSAIA--------------KGVSGIGEAYLSGHQKGTAL        222
Salmonella_phage_Epsilon_15_gp11       QQMAAIRSQG-SGQST--------LGSAI--------------SGGTSGALAGAGIASLL        208
Shigella_phage_Sf6_gp11                QQSAALAAANANKPSS--------LQTAI--------------SGGTSGAIAGAGLASLL        209
Escherichia_phage_CUS-3_gp7-like       QQSAALAAANANRPSA--------LRQGV--------------SGAASGALLGGGIASAL        209
Salmonella_phage_P22_gp7               QQSAALAAANANRPSA--------MQSAI--------------GGGASGAIAGAGLAKLI        208
Salmonella_phage_HK620_gp7             QQSAALAAANANRPSA--------MQSAI--------------GGGASGAIAGAGLAKLI        209
Escherichia_phage_BA14_gp14            QIN-EINKSEPKLKGK--------LEQIIDPLGLGL-------TKLVDVATAGSPLTNKL        181
Klebsiella_phage_K11_gp14              QVK-SMQKAEGKGKSK--------LEQVLDPLALMT-------SQGASAYSSGA-FDSKG        180
Yersenia_phage_phiYeO3-12_gp14         QVD-AMYKSEAKGKSG--------LMRVLDPLSIMG-------QEAASQYAAGG-FDKKG        180
Escherichia_phage_T7_gp14              QID-EIYKSEQKQKSK--------LQMVLDPLAIMG-------SSAASAYASGA-FDSKS        180
```

```
Escherichia_phage_13a_gp14             QID-EIYNSEQKQKSK--------LQMVLDPLAAMG-------SSAASAYASGA-FDSKS        180
                                            :                  :                      .

Prochlorococcus_phage_P-SSP7_gp14      ------GFGWWGG------------       200
Pseudomonas_phage_phiKMV_gp35          ------PKG---------GN-----       181
Escherichia_phage_K1E_gp34             ------GKALTDSRTYSSGTRGI--       240
Salmonella_phage_SP6_gp35              ------SKAWSDSRTYSSGTRGV--       239
Salmonella_phage_Epsilon_15_gp11       GT----STPWGAGIGAGIGLLGSLF       229
Shigella_phage_Sf6_gp11                ET----STPWGAGIGAGIGLLSSLF       230
Escherichia_phage_CUS-3_gp7-like       EL----STPWGAGIGAGLGLLGSLF       230
Salmonella_phage_P22_gp7               GS----STPWGAAIGGGIGLLGSLF       229
Salmonella_phage_HK620_gp7             GS----STPWGAAIGGGLGLLGSLF       230
Escherichia_phage_BA14_gp14            GKKVASKVKASDAKSTGQGK-----       201
Klebsiella_phage_K11_gp14              T--K---APISQAQGTKVGGK----       196
Yersenia_phage_phiYeO3-12_gp14         GNQA---APISAAKGTKTGR-----       197
Escherichia_phage_T7_gp14              TT-K---APIVAAKGTKTGR-----       196
Escherichia_phage_13a_gp14             TT-K---APIVAAKGTKTGR-----       196
```

**Table S2.** Gp15-like sequence alignment.

```
Salmonella_phage_Epsilon_15_gp12       ------------------------------------------------------------        0
Prochlorococcus_phage_P-SSP7_gp15      -------------------------------------------MTNSFFNFTE-------        10
Escherichia_phage_BA14_gp15            -M------------------------------------------SKLAQALGGMNAPSTS        17
Escherichia_phage_T7_gp15              -M------------------------------------------SKIESAL-QAAQPGLS        16
Escherichia_phage_13a_gp15             -M------------------------------------------SKIESAL-QAAQPGLS        16
Klebsiella_phage_K11_gp15              MA------------------------------------------SKLEQALSQLPQAGST        18
Yersenia_phage_phiYeO3-12_gp15         MA------------------------------------------SKLNSVLGNMATPGME        18
Escherichia_phage_K1E_gp35             MAIERQAVQGLP----------QVQATS-PNVMTFAPQQVGG------VEAGVASTSGSR        43
Salmonella_phage_SP6_gp36              MAIERQAVQGLR----------RVQSTGGPSAASFATRQVGV------Q---ETSASGSR        41
Pseudomonas_phage_phiKMV_gp36          MAESQRASQELGINVGQTQLQPGQSARR------------GV--RDSEVNYSG-PSVGSQ        45
Shigella_phage_Sf6_gp12                MATWQG-------SNGGLLAGIGGVNSNAPSVNDIGN-TLQLIRQNNDIERSGANNVGLT        52
Salmonella_phage_P22_gp20              MATWQQ-----GINSGGFLAGIGAQNENAPKARDINA-TLGLIRENNDLARSGANNVALT        54
Salmonella_phage_HK620_gp20            MATWQQ-----GINSGGFLAGIGTQNENAPKASDINA-TLGLIRENNELARSGVNNVGLT        54
Escherichia_phage_CUS-3_gp20-like      MATWQQ-----GINSGGFLAGIGAQNENAPKASDINA-TLGLIRENNELAQSGANNVGLT        54


Salmonella_phage_Epsilon_15_gp12       ------------------------------------------------------------        0
Prochlorococcus_phage_P-SSP7_gp15      ------------------------APDFAAAIGATYESVNTSYDRREELEQKNDAVRLKN        46
Escherichia_phage_BA14_gp15            RLRGTG-RVEVKASTVYEDPKYAKKSALIGTVGKLAEMGADAYM-KYDQHQKDKADERS-        74
Escherichia_phage_T7_gp15              RLRGGAGGMGYRAATTQ--AEQPRS-SLLDTIGRFAKAGADMYT-AKEQRARDLADERS-        71
Escherichia_phage_13a_gp15             RLRGGAGGMGYRAATTQ--AEQPRS-SLLDTIGRFAKAGADMYT-AKEQRARDLADERS-        71
Klebsiella_phage_K11_gp15              RIRGGSASMQYRPVTIQ--QEGFRQSNLVQSLAKFGTAVGEAAD-AYDKRQRDKADERS-        74
Yersenia_phage_phiYeO3-12_gp15         RLRG-VKGMDYRAATIQ--AEQPRA-SLLDSIGRFAKAGADMYM-AKDERDKQRADERS-        72
Escherichia_phage_K1E_gp35             FIEDLI---------------------------RAASSVADVTTGILNQKIEEDKVVQM-        75
Salmonella_phage_SP6_gp36              FLEDLV---------------------------NAAGSLATVTTSILNQRVEDDKVRQY-        73
Pseudomonas_phage_phiKMV_gp36          ILDGIL---------------------------GAGQ--------QI-------AGKWF-        62
Shigella_phage_Sf6_gp12                ALQGLS---------------------------GIAG--------VFQQEKQARRQKEF-        76
Salmonella_phage_P22_gp20              GLRGLA---------------------------GVAD--------IYNQEQQQKALNAF-        78
Salmonella_phage_HK620_gp20            ALRGLA---------------------------GVAD--------IYNQEQQQKAISAF-        78
Escherichia_phage_CUS-3_gp20-like      ALRGLA---------------------------GVAD--------IYKQDQQQKAINAF-        78


Salmonella_phage_Epsilon_15_gp12       -----------------------------------------------------MELDAIL        7
Prochlorococcus_phage_P-SSP7_gp15      AETLEKLPEEII-KTLPTVKKAVDAINKKRREALL-----------------AKGYEGID        88
Escherichia_phage_BA14_gp15            --------NEIIRKLTPEQR----------------------------------------        86
Escherichia_phage_T7_gp15              --------NEIIRKLTPEQR----------------------------------------        83
Escherichia_phage_13a_gp15             --------NEIIRKLTPEQR----------------------------------------        83
Klebsiella_phage_K11_gp15              --------DEIIRKLTPEQR----------------------------------------        86
Yersenia_phage_phiYeO3-12_gp15         --------NEIIRKLTPEQR----------------------------------------        84
Escherichia_phage_K1E_gp35             --------ERAYNGLMPSEDA----TRGGARANMLVKAQLLANDEAARMKDMATRFQGTD        123
Salmonella_phage_SP6_gp36              --------NRALTGLMPTEDA----TVGGARAHMLVSLQNDIIAQTMQLSDDAQRFDGDD        121
Pseudomonas_phage_phiKMV_gp36          --------EHNV------QQE----VLRGERARMAGEAE---E----AVDSNVLAKPFVK        97
Shigella_phage_Sf6_gp12                --------QQAY------ANA----YASGDRGALRQLATQYPD----QIESVRKGMGFI-        113
Salmonella_phage_P22_gp20              --------NQVH------ANA----WATGDPSGLFKFAQENPA----FVAQAQQAFSGL-        115
Salmonella_phage_HK620_gp20            --------NKVH------ADA----WASGDPSGLFKFAQENPA----FVAQAQQAFSGL-        115
Escherichia_phage_CUS-3_gp20-like      --------NKVH------ADA----WASGDPSGLFKFAKENPA----FVAQAQQAFSGL-        115


Salmonella_phage_Epsilon_15_gp12       ----DNLSDEEQ--IELLELLEEEENYRN-------------------------------        30
Prochlorococcus_phage_P-SSP7_gp15      ----QELIDKDKESLNTLFNIGKAENFIKNEALKNGDNVTYETIDLSGPH-GTRR-----        138
Escherichia_phage_BA14_gp15            ---REAI-----KNGTLLYQD--DPYAMEALKTKTGRNAAYLV-----------------        119
Escherichia_phage_T7_gp15              ---REAL-----NNGTLLYQD--DPYAMEALRVKTGRNAAYLV-----------------        116
Escherichia_phage_13a_gp15             ---REAL-----NNGTLLYQD--DPYAMEALRVKTGRNAAYLV-----------------        116
Klebsiella_phage_K11_gp15              ---REAI-----KNGTLLYQD--DPYAMEALRFKTGRNAAFLI-----------------        119
Yersenia_phage_phiYeO3-12_gp15         ---RQAI-----QNGTLLYQD--DPYAMQALKFKTGRNAAFLV-----------------        117
Escherichia_phage_K1E_gp35             DEWTQLMVDSRNEMQNKLFQQ--YPELQGDK--DTMRMVTNVFQEQQPQIWATRTQHKLD        179
Salmonella_phage_SP6_gp36              SQWEDHVINARMAVQDRLWDT--YPELRGDK--ESMRVVTNAFMEQQPKIFAARETAKLK        177
Pseudomonas_phage_phiKMV_gp36          GGWRKQ--------------D--YRIAQADFSLKMQRFIANKGREM--------------        127
Shigella_phage_Sf6_gp12                ----DE--------------D--QRNSIGTL-----AAGARLAA-S--------------        133
Salmonella_phage_P22_gp20              ----NE--------------Q--QRNDMGDL-----AMKANVALSQ--------------        136
Salmonella_phage_HK620_gp20            ----NE--------------Q--QRNDMGDL-----AMRANVALSQ--------------        136
Escherichia_phage_CUS-3_gp20-like      ----NE--------------Q--QRNDMGDL-----AMRANVALSQ--------------        136
                                           .
```

```
Salmonella_phage_Epsilon_15_gp12         -----------TH-------------------------------LLYEFAPYSK------        42
Prochlorococcus_phage_P-SSP7_gp15        --RLLMIEEIKQR-------------------------------LATEFNPWVTKNYPAG        165
Escherichia_phage_BA14_gp15              ---------------------------DDEVAQKVKNGEFRTRQELEEFRHSRLQEAAKN        152
Escherichia_phage_T7_gp15                ---------------------------DDDVMQKIKEGVFRTREEMEEYRHSRLQEGAKV        149
Escherichia_phage_13a_gp15               ---------------------------DDDVMQKIKEGVFRTREEMEEYRHSRLQEGAKA        149
Klebsiella_phage_K11_gp15                ---------------------------DDEVAQRVQNGEFRTRAEMEEYRHKRLTEGANE        152
Yersenia_phage_phiYeO3-12_gp15           ---------------------------DDEVQQAIKEGRFRTREEMEQYRHSRLQEHSKS        150
Escherichia_phage_K1E_gp35               REQADREDTFDGRVASTWDPNIDPEASGYALQERIREGLT-QGLLPEQMHKKLVQRAISL        238
Salmonella_phage_SP6_gp36                QEAEARIKSMESRILLAT-RDVPGEAMGDALNQLQKEAMA-MQITKQEFDALVSQLAANR        235
Pseudomonas_phage_phiKMV_gp36            --------------------------------------------TPEEFRKYLSQEATHV        143
Shigella_phage_Sf6_gp12                  --------------------------------------------SPEAMQSWLQNNANEL        149
Salmonella_phage_P22_gp20                --------------------------------------------GPEAYSKFITDNKDRL        152
Salmonella_phage_HK620_gp20              --------------------------------------------GPEAYSKFITDNKDRL        152
Escherichia_phage_CUS-3_gp20-like        --------------------------------------------GPEAYSKFITDNKDRL        152

Salmonella_phage_Epsilon_15_gp12         ------------------------------------------------------------        42
Prochlorococcus_phage_P-SSP7_gp15        FNTVQEARLAFDKYKQGILNNADELGFNLRFTKSQLKDSFNT--------VESTFYETTN        217
Escherichia_phage_BA14_gp15              YAEQFGIDETDEHYQKGFNSDITERNIALYGAHDNFLSDQAKKGAVINSRVEL-------        205
Escherichia_phage_T7_gp15                YAEQFGIDPEDVDYQRGFNGDITERNISLYGAHDNFLSQQAQKGAIMNSRVEL-------        202
Escherichia_phage_13a_gp15               YAEQFGIDPEDVDYQRGFNGDITERNISLYGAHDNFLSQQAQKGAIMNSRVEL-------        202
Klebsiella_phage_K11_gp15                FAEQFMINPEDSEFQRGFNANITERNISLYGKHDTFLSEQAQKGAILASKVEL-------        205
Yersenia_phage_phiYeO3-12_gp15           FAEQFGINEMDEEYQKGFNANITERNIALYGAHDTFLSDQAQKGAILNSKVEL-------        203
Escherichia_phage_K1E_gp35               AQGG----D--VSMAEALKYVKDDKGVSVYAKNPQLITAITSGNAVWARNNVADVTRMSF        292
Salmonella_phage_SP6_gp36                AAIG----D--DSMIQGTKSLKDENGVSLYDRVGQLQTGEIQANRTWAAQNQVALFEKKD        289
Pseudomonas_phage_phiKMV_gp36            LDSTEGMNP--NDALQALAQQQ--------KAEEQLFGMQAKAYMDWSIDQAARGFRTQG        193
Shigella_phage_Sf6_gp12                  A--RVGVDP--NNVAQMYQQNP--------SGFGEFV---DHLG--M----AAL------        182
Salmonella_phage_P22_gp20                N--RVGANP--DWMIQTGVQNP--------EQLSHML---TTMS--L----GAL------        185
Salmonella_phage_HK620_gp20              N--RVGANA--DWMIQTGIQNP--------EQLSHML---TTMS--L----GAL------        185
Escherichia_phage_CUS-3_gp20-like        N--RVGANA--DWMIQTGIQNP--------EQLSHML---TTMS--L----GAL------        185

Salmonella_phage_Epsilon_15_gp12         ----------------------------QREFID----------A-----GHDYPE----        55
Prochlorococcus_phage_P-SSP7_gp15        TAITSKNVLKEQSRMISEVTHALNAEDPLKAFIEASEYNIGYFEG-----NIAKAE----        268
Escherichia_phage_BA14_gp15              ----------------N---SVLNDPE-TLRSPHAGEFFENYFSAGLTTGSIPSDD---Q        242
Escherichia_phage_T7_gp15                ----------------N---GVLQDPD-MLRRPDSADFFEKYIDNGLVTGAIPSDA---Q        239
Escherichia_phage_13a_gp15               ----------------N---GVLQDPD-MLRRPDSADFFEKYIDNGLVTGAIPSDA---Q        239
Klebsiella_phage_K11_gp15                ----------------S---GVLKDPA-VLARPESGEFFQRYIDNALKTGSIPSDA---Q        242
Yersenia_phage_phiYeO3-12_gp15           ----------------N---GVLSDPD-LLSRPESGEFFQNYIDNSLVTGMT--DN---Q        238
Escherichia_phage_K1E_gp35               EVKESY---------LA---GDLTD-----------------------------------        305
Salmonella_phage_SP6_gp36                AAIKAF---------EA---GQLNR-----------------------------------        302
Pseudomonas_phage_phiKMV_gp36            NSILAKAV---QAQATG---DELSR---QLSLEEAGLFYTNIMT----SEDIPLEVRDKV        240
Shigella_phage_Sf6_gp12                  GPIDYFNV---QDKMAG---REIDR---GRLAET-------IR-----S--------NKA        213
Salmonella_phage_P22_gp20                GPEKAFAV---QDKMVG---REIDR---GRLAET-------IR-----S--------NKA        216
Salmonella_phage_HK620_gp20              GPEKAFAV---QDKMVG---RQQEQ---QRINET-------IR-----N--------NDM        216
Escherichia_phage_CUS-3_gp20-like        GPEKAFAV---QDKMVG---RQQEQ---QRINET-------IR-----N--------NDM        216

Salmonella_phage_Epsilon_15_gp12         -RCFMAG----NQLG---KSFTG---AA--EVAFHLTGRY--PGTKGYPADGKYGGEWKG        100
Prochlorococcus_phage_P-SSP7_gp15        -RAFINI----GLMG---TKKGS---INIDKFESVLFGEVTAKGDKTRILIDKLGGGE--        315
Escherichia_phage_BA14_gp15              AFTM--ISQGLSDV--VNREGGGQFLQQIENRKVKLHGKETT-------F-KELMGAEQW        290
Escherichia_phage_T7_gp15                ATQL--ISQAFSDA--SSRAGGADFLMRVGDKKVTLNGATTT-------Y-RELIGEEQW        287
Escherichia_phage_13a_gp15               ATQL--ISQAFSDA--SSRAGGADFLMRVGDKKVTLNGATTT-------Y-RELMGEEQW        287
Klebsiella_phage_K11_gp15                AQQV--IIGSLNDV--IQRPGATNFLQSLEGRPVTLNGKTTT-------Y-KELMGEEQW        290
Yersenia_phage_phiYeO3-12_gp15           AQQV--ISSSLNDV--LQRPGGAAFLQNIENRKVTLNGATTT-------Y-RELMGDEQW        286
Escherichia_phage_K1E_gp35               -EELLERAQHINNL-----TGNSVFSNP--------------------------------        327
Salmonella_phage_SP6_gp36                -EQLLQVMQNHNEI-----SGGTAWSDS--------------------------------        324
Pseudomonas_phage_phiKMV_gp36            GMQFLAASLDMNQRGIYEGLRDAGFLDS-MSFD---------------------------        272
Shigella_phage_Sf6_gp12                  GEALTVRGQDIQIRGQNISAQNAALSREIQRAE---------------------------        246
Salmonella_phage_P22_gp20                GEGLQARGQNITMRGQDMSAATARRGQDLATQR---------------------------        249
Salmonella_phage_HK620_gp20              TNARAIRGQDLSYKAQMARLNH-----DKYVFK---------------------------        244
Escherichia_phage_CUS-3_gp20-like        TNARAIRGQDLSYKAQMARLNH-----DKYVFK---------------------------        244

Salmonella_phage_Epsilon_15_gp12         ---------------KRFYEPVVFWIGGETNETV--------------------------        119
Prochlorococcus_phage_P-SSP7_gp15        --------------------ENRLWAEGVLNEIENAKKGVFENKELNRTNYA---KGF--        350
Escherichia_phage_BA14_gp15              NNLMVKAQHNEFQLNAKKTEAFQLNVNSALNQ-ENVNTGWEQLQSIKAELDTLQPGE---        346
Escherichia_phage_T7_gp15                NALMVTAQRSQFETDAKLNEQYRLKINSALNQ-EDPRTAWEMLQGIKAELDKVQPDE---        343
Escherichia_phage_13a_gp15               NALMVTAQRSQFENDAKLNEQYRLKINSALNQ-EDPRTSWEMLQGIKAELDKVQPDE---        343
Klebsiella_phage_K11_gp15                NALMVKAQSTQFDNDAKLSEGFRLGITSALNQ-DDTSKGWEMLQGAKAELDRLQPGE---        346
Yersenia_phage_phiYeO3-12_gp15           QAMMIKAQHTQFQNNAKLTEKFRLDINSALNQ-ADTGKGWETLQGIKAELDKIQPGE---        342
Escherichia_phage_K1E_gp35               ------------------------ELEALMRQRAKQNAELGAMQDMRRELYSDRLTGFQG        363
Salmonella_phage_SP6_gp36                ------------------------EIKSLFDRQAKARATSAKLEDLVARGEHGSPLGLQD        360
Pseudomonas_phage_phiKMV_gp36            --------------D-------RRALNGLYEKSKAQTRAKESMATLRA-D-----ADF--        303
Shigella_phage_Sf6_gp12                  -------------LQ-------EKALDRQIARESNQLK----LEELKQKQ-----ADV--        275
Salmonella_phage_P22_gp20                -------------AN-------ARTISGSEGNRVVQL------ADGRT-------VSV--        274
Salmonella_phage_HK620_gp20              -------------QS------------QAALERAGQL------QDMDV-------LSL--        264
Escherichia_phage_CUS-3_gp20-like        -------------QS------------QAALERAGQL------QDMDV-------LSL--        264
                                                                        .

Salmonella_phage_Epsilon_15_gp12         ---------------------------------------------TKTTQRILCGRIEEN        134
Prochlorococcus_phage_P-SSP7_gp15        ---------VEGI---------------------QEEENKTN---TRMTKVELARYVTEN        377
Escherichia_phage_BA14_gp15              EMTPERQALINAQTQMQDRMKRETAELAKQMDKQQKSMNKMNVIDAQFQKRLNGQYVSTA        406
Escherichia_phage_T7_gp15                QMTPQREWLISAQEQVQNQMNAWTKAQAKALDDSMKSMNKLDVIDKQFQKRINGEWVSTD        403
```

```
Escherichia_phage_13a_gp15            QMTPQREWLISAQEQVQNQMNAWTKAQAKALDDSMKSMNKLDVIDKQFQKRINGEWVSTD   403
Klebsiella_phage_K11_gp15             QMTPERERLIQAEEQMQARFRQEAQAAAKEMDKRQKTINKNQVIDQQFTKRINGQYVSTS   406
Yersenia_phage_phiYeO3-12_gp15        EMTPEREWLISAQEQMRTRFKQEQAETAKLMDKQQKTLNKSLVIDKQFQKRLNGEYVSTN   402
Escherichia_phage_K1E_gp35            KTDKEKKAYIDVIKQDSQLYAD------------QQIKQRGLDPYSQEAEAIRGAVEVQR   411
Salmonella_phage_SP6_gp36             ISKEDRKAYAGALVDAYTKLAN------------DEITRTG--ATGEEAEAIRGRYEQMR   406
Pseudomonas_phage_phiKMV_gp36         ---------------------Q------------QRVANGAITDLAEVEAYSRGMVEEGR   330
Shigella_phage_Sf6_gp12               ---------------------R------------QKADIARADR---QAAA---QGAVDT   296
Salmonella_phage_P22_gp20             ---------------------G------------GKLHGAGANA----------------   285
Salmonella_phage_HK620_gp20           ---------------------N------------SQIAATGIDPLTGKAATSARMSQAKR   291
Escherichia_phage_CUS-3_gp20-like     ---------------------N------------SQIAATGIDPLTGKAATSARMSQAKR   291


Salmonella_phage_Epsilon_15_gp12      DEPGYGSIPKEDIISWKKSPFFPNLVDHLLVKHHTP-EGVEDGISICYF------KP---   184
Prochlorococcus_phage_P-SSP7_gp15     WDITQGG---SNLPEFVKNRFAKETGDDILIKAELD-YKLDKGIPITEKEVLKLSDPFVE   433
Escherichia_phage_BA14_gp15           YGDMPTN---ENTGEFTHSDMV--------------NYANKKLADIDAMDIPAEQKDRMK   449
Escherichia_phage_T7_gp15             FKDMPVN---ENTGEFKHSDMV--------------NYANKKLAEIDSMDIPDGAKDAMK   446
Escherichia_phage_13a_gp15            FKDMPVN---ENTGEFKHSDMV--------------NYANKKLAEIDSMDIPDAAKDAMK   446
Klebsiella_phage_K11_gp15             YKDMPTN---ENTGEFTHSDMV--------------NYANGKLAEIDQMQLTEQQKDRMK   449
Yersenia_phage_phiYeO3-12_gp15        YKDMPTN---ENTGEFTHSDMV--------------NYANQKLAEIEAMDLTPEQKDKLK   445
Escherichia_phage_K1E_gp35            LQFMN-------------SKGL---VDDTF-ESRIK-AMES-MLS-PEHFAKGEPQ---E   448
Salmonella_phage_SP6_gp36             YAKLG-------------QQLI---EDPII-KERYG-SLMQ-LSSANLKDMKIEPE---A   444
Pseudomonas_phage_phiKMV_gp36         WSDAQAI------------------------------SFMT-----KAMTGLGNAQ---R   352
Shigella_phage_Sf6_gp12               FSTALDS---LNEIE--------------------------------QSPGLSKAV---G   318
Salmonella_phage_P22_gp20             FYEGIDD---NGNMVRVPASAI---AAPPTSAASAQNYAMK-----KDIDAIANAD---A   331
Salmonella_phage_HK620_gp20           WLDGNNN---YNNALITGERGI----------EKIDSLLGK-----KELEGIGRFE---G   330
Escherichia_phage_CUS-3_gp20-like     WLDGNNN---YNNALITGERGI----------EKIDSLLGK-----KELEGIGRFE---G   330


Salmonella_phage_Epsilon_15_gp12      -----------------------------YSQG------------RARWQGDT-------   196
Prochlorococcus_phage_P-SSP7_gp15     AQYLAKVKTGNPLAPSSDFKALAKSQIKGYATVHAKQAGVAPGKESPQWNNIVENAEREY   493
Escherichia_phage_BA14_gp15           LDYLKADSEKG-------------------A--FRTAVGELIGDAEKEWTSA--------   480
Escherichia_phage_T7_gp15             LKYLQADSKDG-------------------A--FRTAIGTMVTDAGQEWSAA--------   477
Escherichia_phage_13a_gp15            LKYLQADSKDG-------------------A--FRTAIGTMVTDAGQEWSAA--------   477
Klebsiella_phage_K11_gp15             LSYLRADSEGG-------------------A--FRTVVGQLVTDAGSEWSAA--------   480
Yersenia_phage_phiYeO3-12_gp15        LDYLRADSKEG-------------------A--FRAAFGEMITDAGNEWQAA--------   476
Escherichia_phage_K1E_gp35            LMT-----------------------------------------IRQLWEQLPEE-SRGV   466
Salmonella_phage_SP6_gp36             LQT-----------------------------------------IMRARDSIPEDARRAV   463
Pseudomonas_phage_phiKMV_gp36         MQGIMAALEAG-------------------DINALHTLGTNVTEALEQWDKMQAANGSSL   393
Shigella_phage_Sf6_gp12               -------------------------------------------IRSA-------------   322
Salmonella_phage_P22_gp20             -------------------------------------------SALDFMTGMTGGAGNP-   347
Salmonella_phage_HK620_gp20           -------------------------------------------RNIDGFTSAEGLANRN-   346
Escherichia_phage_CUS-3_gp20-like     -------------------------------------------RNIDGFTSAEGLANRN-   346


Salmonella_phage_Epsilon_15_gp12      ---------------------------------------I--------------------   197
Prochlorococcus_phage_P-SSP7_gp15     P----------LLFAKYMQTADSAVDAHILALKDLESRTNNGVYDNLVVNTDKNKIRSVE   543
Escherichia_phage_BA14_gp15           -----------VINGKMPEDGGVALN------A---LRR--------VRNADPELFAALY   512
Escherichia_phage_T7_gp15             -----------VINGKLPERT-PAMD------A---LRR--------IRNADPQLIAALY   508
Escherichia_phage_13a_gp15            -----------VINGKLPERT-PAMD------A---LRR--------IRNADPQLIAALY   508
Klebsiella_phage_K11_gp15             -----------VINGKLPEDT-TALN------K---LRT--------MRNTDPDLFAALY   511
Yersenia_phage_phiYeO3-12_gp15        -----------VINGKMPEST-PAMD------N---LRR--------IRNTDPDLIASLY   507
Escherichia_phage_K1E_gp35            ------------------------------------------------------------   466
Salmonella_phage_SP6_gp36             ------------------------------------------------------------   463
Pseudomonas_phage_phiKMV_gp36         TDRLVQGTQLGLRLGTFPKTYGESVGSAVRMIQ---AAK--------EGEANPELVNTLN   442
Shigella_phage_Sf6_gp12               ----------------FPTVPGSD---------------------------AANFEARLD   339
Salmonella_phage_P22_gp20             ----------AIGADVRSRLTGKE---------------------------QRQLYNSAQ   370
Salmonella_phage_HK620_gp20           ----------AI-----------------------------------------------E   349
Escherichia_phage_CUS-3_gp20-like     ----------AI-----------------------------------------------E   349


Salmonella_phage_Epsilon_15_gp12      -----------------------HGVWFDEEPPYS------------IYG------EGLT   216
Prochlorococcus_phage_P-SSP7_gp15     LIKAEEH------IKTIDPNVINTGLIFGTEEVIKEAAELPTGQTHLFYDQLAAKIPGVT   597
Escherichia_phage_BA14_gp15           PDKAEMFLTMDM----MDNQGIDPQILLDADKARQS---------------LTKEMQYE-   552
Escherichia_phage_T7_gp15             PDQAELFLTMDM----MDKQGIDPQVILDADRLTVK---------------RSKEQRFE-   548
Escherichia_phage_13a_gp15            PDQAELFLTMDM----MDKQGIDPQVILDADRLTAK---------------RSKEQRFE-   548
Klebsiella_phage_K11_gp15             PDKADLFLTMDM----MDKQGIDPQILIDADRSRRS---------------LTKEMQYE-   551
Yersenia_phage_phiYeO3-12_gp15        PDKAELFLTMDM----LDNQGIDTQVLIDADKSRAQ---------------KTKEMQFE-   547
Escherichia_phage_K1E_gp35            ----------------------------------------------------------F-   467
Salmonella_phage_SP6_gp36             ----------------------------------------------------------M-   464
Pseudomonas_phage_phiKMV_gp36         SI----F-EQVASAQEIN-----P-SA------GNV---------------M---LSGI-   466
Shigella_phage_Sf6_gp12               TFKAQTFLPMVQSLKGMGALS---DAE------GKK---------------L---SDAV-   371
Salmonella_phage_P22_gp20             RIQGRMQNQGVAAARDMGASGINTIAE------AKM---------------Y---FQGM-   405
Salmonella_phage_HK620_gp20           ELKSGAFVQNVQTMRGMGSLS---NAE------GQK---------------L---ENLI-   381
Escherichia_phage_CUS-3_gp20-like     ELKSGAFVQNVQIMRGMGSLS---NAE------GQK---------------L---ENLI-   381


Salmonella_phage_Epsilon_15_gp12      R-TNKYGQFSILTFT-------------PLMGMSDVVTKFLKNPSKSQKVVNMTIYDAEH   262
Prochlorococcus_phage_P-SSP7_gp15     GAEIQYKQVEIYNKMNKLEKPVKSDVLLAYEKLSPTVQFYLSHHPSPAKVARAKI---EA   654
Escherichia_phage_BA14_gp15           -DDKAWASLKNNSE-----------------------SPELSRMPATLDAGARKIYDSVK   588
Escherichia_phage_T7_gp15             -DDKAFESALNASK-----------------------APEIARMPASLRESARKIYDSVK   584
Escherichia_phage_13a_gp15            -DDKAFESALNSST-----------------------APEIARMPASLRESARKIYDSVK   584
Klebsiella_phage_K11_gp15             -DDKAWASLKNNSE-----------------------SPELSRIPASLDGMARKIYDSVK   587
Yersenia_phage_phiYeO3-12_gp15        -DDRAWAAMMNDSK-----------------------NPQIKYMPSSVQGYARKIYDSVK   583
Escherichia_phage_K1E_gp35            -GDTVNGYMDNYNT-----------------------ALQMGETPLQAARFAR-------   496
Salmonella_phage_SP6_gp36             -GDKEYAFAENYDL-----------------------ATRMGYTPGQAIEFAQ-------   493
```

```
Pseudomonas_phage_phiKMV_gp36        -PEAEQGAVAWALK-----------------------QMKMGIAPAQALR---------E    493
Shigella_phage_Sf6_gp12              -GALSPKMSEKAFR-----------------------------DSIGKIRNQLES----K    397
Salmonella_phage_P22_gp20            -PQVDYSSPEAMQ------------------------------QSIREIQEYTNNYNQ-Q    433
Salmonella_phage_HK620_gp20          -AKLDITQPEEVVR-----------------------------KQLSEIR---SQYSV-F    407
Escherichia_phage_CUS-3_gp20-like    -AKLDITQPEEVVR-----------------------------KQLSEIR---SQYSV-F    407


Salmonella_phage_Epsilon_15_gp12     YT-DEQK---EQIIASYPEHEREARARGIPTMGSG-------------------------    293
Prochlorococcus_phage_P-SSP7_gp15    FK-DDAQIDFDEIDFLLP-AAKEALEKTIEDEGAS-------------------------    687
Escherichia_phage_BA14_gp15          YRTGNADMAMQQVDKYLKENTTTLTGDGVDGDTIGVLTRNALRVTDDPDSWKQ-------    641
Escherichia_phage_T7_gp15            YRSGNESMAMEQMTKFLKESTYTFTGDDVDGDTVGVIPKNMMQVNSDPKSWEQ-------    637
Escherichia_phage_13a_gp15           YRSGNESMAMEQMTKFLQESTYTFTGDDVDGDTVGVIPKNMMQVNSDPKSWEQ-------    637
Klebsiella_phage_K11_gp15            YRTGNSDMAMQQTDKFLKESTVTFKGDDVDGDTIGIIPKNILQVSDDPKSWEQ-------    640
Yersenia_phage_phiYeO3-12_gp15       YRTGNPDMATEQVAKFLSDSTTTFSSSDVDGDTYGVLPKNILTVSDDPKSWEQ-------    636
Escherichia_phage_K1E_gp35           ----EA-----QQKFSRTEKETKKFNSAIGDALDE----------VSGAGWFD--GKTE-    534
Salmonella_phage_SP6_gp36            ----NA-----SRGDKLPGSVMKELNDEVDGVVSD----------VASGSWLT--RGDN-    531
Pseudomonas_phage_phiKMV_gp36        F-SANAE-VVKQMDEFEKGQNTKAFKDNLGKQVNDKFVNN-----IFGRAWNMLTGESDL    546
Shigella_phage_Sf6_gp12              L-S---T-VKKQFDYQEPVQNTPVQ-----Q-----------------------------    418
Salmonella_phage_P22_gp20            Y-NVN----VGNGGLKSPRQQPDTQQSAGGS-----------------------------    459
Salmonella_phage_HK620_gp20          Q-KVAAR-EAESMGYSSSGYDTYVSERKSGS-----------------------------    436
Escherichia_phage_CUS-3_gp20-like    Q-KVAAR-EAESMGYSSSGYDTYVSERKSGS-----------------------------    436
                                                .

Salmonella_phage_Epsilon_15_gp12     ------------RIFQI-------PE-E-----T--------------------------    302
Prochlorococcus_phage_P-SSP7_gp15    ------------FILNE-------LDTEEFLELD--------------------------    702
Escherichia_phage_BA14_gp15          ---------GKDII-------------------DTAAK----------------------    651
Escherichia_phage_T7_gp15            ---------GRDIL-------------------EEARK----------------------    647
Escherichia_phage_13a_gp15           ---------GRDIL-------------------EEARK----------------------    647
Klebsiella_phage_K11_gp15            ---------GRDIL-------------------EEARK----------------------    650
Yersenia_phage_phiYeO3-12_gp15       ---------GKNIL-------------------DEAIK----------------------    646
Escherichia_phage_K1E_gp35           -----VSDLGKAIAEEELRAKANMLWSSGMRNMDSIKKALITWGNKRYTQSEDAKTSGGY    589
Salmonella_phage_SP6_gp36            -----MSDMGRDLMLEEANQIARSMKVAG-HNNDTIKRHLKSFLQNQYTQLSEGFFTQGV    585
Pseudomonas_phage_phiKMV_gp36        SNNEAVLSMYRRATIDEANWLASDRKHAGLLTSDTGREALLEIAAANVRNRTIQVGEGRN    606
Shigella_phage_Sf6_gp12              ------------------------------------------------------------    418
Salmonella_phage_P22_gp20            ------------------------------------------------------------    459
Salmonella_phage_HK620_gp20          ------------------------------------------------------------    436
Escherichia_phage_CUS-3_gp20-like    ------------------------------------------------------------    436


Salmonella_phage_Epsilon_15_gp12     ---------IKCQP----------------------------------------------    307
Prochlorococcus_phage_P-SSP7_gp15    ---------TEAQE----------------------------------------------    707
Escherichia_phage_BA14_gp15          ------------------------KLAETNPWVTNKQLTVFERGDSIYLMD------TTG    681
Escherichia_phage_T7_gp15            ------------------------GIIASNPWITNKQLTMYSQGDSIYLMD------TTG    677
Escherichia_phage_13a_gp15           ------------------------GIIASNPWITNKQLTMYSQGDSIYLMD------TTG    677
Klebsiella_phage_K11_gp15            ------------------------GIIAANPWVTNKQLTMYQQGDSIYMMD------TTG    680
Yersenia_phage_phiYeO3-12_gp15       ------------------------RITEANPWITNKQLTVYQQGDAIYLMD------TTG    676
Escherichia_phage_K1E_gp35           FIKGDYTSASD---MLMSVG------KGVNPTDVPLALGRYVETQMPELKKELQEWETKD    640
Salmonella_phage_SP6_gp36            LVKGDVRT--------LGDT------IGANQKDVPTVLRQYLDNHKQALLDASG-GMEEG    630
Pseudomonas_phage_phiKMV_gp36        LKEGDLFSRRDSAPLILPRGTTAEQLFGTND--TETIGTVLAEQHKPHVEGLLG---YKS    661
Shigella_phage_Sf6_gp12              ------------------------------------------------------------    418
Salmonella_phage_P22_gp20            ------------------------------------------------------------    459
Salmonella_phage_HK620_gp20          ------------------------------------------------------------    436
Escherichia_phage_CUS-3_gp20-like    ------------------------------------------------------------    436


Salmonella_phage_Epsilon_15_gp12     ------------------------------------------------------------    307
Prochlorococcus_phage_P-SSP7_gp15    ------------------------------------------------------------    707
Escherichia_phage_BA14_gp15          QVNIRYDKQ---LLSKMYQE--------------NQAKLDEEARNKALKDANKRTLHTRA    724
Escherichia_phage_T7_gp15            QVRVRYDKE---LLSKVWSE--------------NQKKLEEKAREKALADVNKRAPIVAA    720
Escherichia_phage_13a_gp15           QVRVRYDKE---LLSKVWSE--------------NQKKLEEKAREKALADVNKRAPIVAA    720
Klebsiella_phage_K11_gp15            TVRIRYDKE---LLTRTYQE--------------QQQRLAKEAEEKALKEATKRAPIAAA    723
Yersenia_phage_phiYeO3-12_gp15       QVRTRFDQE---LLKREYQR--------------TAQMQAEAAREKALKEANKRAPISRV    719
Escherichia_phage_K1E_gp35           DVYIDYNEQKGTFVIR-AGAAGRPLSGVIPVTSLDTTSLLDSAYQKKVEERDKGEYVH--    697
Salmonella_phage_SP6_gp36            DLYFDVDSKRGMFTIR-AGSGRVPVTPAMPLSEIKGQDLLKEHYEKAVKERDEAKKNFEA    689
Pseudomonas_phage_phiKMV_gp36        VVAFEYDRTSGSLLAVEYDENGVAL---------DRTRVDPQAVGKEVLKRNADK-----    707
Shigella_phage_Sf6_gp12              ------------------------------------------------------------    418
Salmonella_phage_P22_gp20            ------------------------------------------------------------    459
Salmonella_phage_HK620_gp20          ------------------------------------------------------------    436
Escherichia_phage_CUS-3_gp20-like    ------------------------------------------------------------    436


Salmonella_phage_Epsilon_15_gp12     ------------------------------------------------FECP----DHFY    315
Prochlorococcus_phage_P-SSP7_gp15    ------------------------------------------------LIMPTPQLGEYN    719
Escherichia_phage_BA14_gp15          MNRKR-----EREAKKPKRSGSMYDSVSGKGILDTLTGKD--------------------    759
Escherichia_phage_T7_gp15            TKAREAAAKRVREKRK-QTPKFIYGRKE--------------------------------    747
Escherichia_phage_13a_gp15           TKAREAAAKLVRDKRK-QTPKFIYGRKE--------------------------------    747
Klebsiella_phage_K11_gp15            TQARKAAGERVRAKRK-ATPKFIYGGGDQ-------------------------------    751
Yersenia_phage_phiYeO3-12_gp15       PQAREEARKRVQEKRK-KTPKYIYGRKED-------------------------------    747
Escherichia_phage_K1E_gp35           ---P-----------------YRTDIGAQEPMPAKPTAKDIGKLGLANFLMSSAFASGEN    737
Salmonella_phage_SP6_gp36            NQMR-----------------MWGAGGYQSPAPEKTTAKTVGSRGIADFLMSPAFASGEN    732
Pseudomonas_phage_phiKMV_gp36        ---------------------LNAMRGAEYGANVKVSGTD--------IRMNGGNS--AG    736
Shigella_phage_Sf6_gp12              ------------------------------------------------------------    418
Salmonella_phage_P22_gp20            ------------------------------------------------------------    459
Salmonella_phage_HK620_gp20          ------------------------------------------------------------    436
Escherichia_phage_CUS-3_gp20-like    ------------------------------------------------------------    436
```

```
Salmonella_phage_Epsilon_15_gp12     VIDAQDFGWNHPQAHIQLWWDKDADVFYLARVWKKSE-----------------------     352
Prochlorococcus_phage_P-SSP7_gp15    RAEPVEGNWQKIPNQIGY-------YVYKNGEWQKSGQRGKNKLEYKDLIDGYRDIDGYF     772
Escherichia_phage_BA14_gp15          ------------------------------------------------------------     759
Escherichia_phage_T7_gp15            ------------------------------------------------------------     747
Escherichia_phage_13a_gp15           ------------------------------------------------------------     747
Klebsiella_phage_K11_gp15            ------------------------------------------------------------     751
Yersenia_phage_phiYeO3-12_gp15       ------------------------------------------------------------     747
Escherichia_phage_K1E_gp35           LPSNFEINYRGNMQQF-----------YDKLAMDENK-----------DKVGFNKATGTF     775
Salmonella_phage_SP6_gp36            LPSNFEFNYKRNNMDF-----------YNYVAKTENG-----------ANVGFDRVAGVY     770
Pseudomonas_phage_phiKMV_gp36        MLKQDVFNWRKELAQF-----------EAY----------------------------RG     757
Shigella_phage_Sf6_gp12              ------------------------------------------------------------     418
Salmonella_phage_P22_gp20            ------------------------------------------------------------     459
Salmonella_phage_HK620_gp20          ------------------------------------------------------------     436
Escherichia_phage_CUS-3_gp20-like    ------------------------------------------------------------     436

Salmonella_phage_Epsilon_15_gp12     ---------NTAVQAWGAVKSWANK---IPVAWPHDGHQHEKGGGEQLKTQYAD-----A     395
Prochlorococcus_phage_P-SSP7_gp15    KPTFKADPSNPNLGAWKKIPNAIGYFVWDGKEWVRSGQ---KGK----------------     813
Escherichia_phage_BA14_gp15          ------------------------------------------------------------     759
Escherichia_phage_T7_gp15            ------------------------------------------------------------     747
Escherichia_phage_13a_gp15           ------------------------------------------------------------     747
Klebsiella_phage_K11_gp15            ------------------------------------------------------------     751
Yersenia_phage_phiYeO3-12_gp15       ------------------------------------------------------------     747
Escherichia_phage_K1E_gp35           TPYKDAHGESIGYGHFLTEEEKRDGYIKIGDELVPY-----RGSMSQLTESKARALMEQD     830
Salmonella_phage_SP6_gp36            TPYKDAHGQSVGYGHFLTEEEKKNGYITIGEDKVPF-----APGQSQLTPERAMRLLEQD     825
Pseudomonas_phage_phiKMV_gp36        EAYKDADGYSVGLGHYLGSGNAGAGTTV--------------------TPEQAAQWFAED     797
Shigella_phage_Sf6_gp12              ------------------------------------------------------------     418
Salmonella_phage_P22_gp20            ------------------------------------------------------------     459
Salmonella_phage_HK620_gp20          ------------------------------------------------------------     436
Escherichia_phage_CUS-3_gp20-like    ------------------------------------------------------------     436

Salmonella_phage_Epsilon_15_gp12     GFSMLPEHATFPD-----------G--G---NSVESGISELRDLMLEG------RFKVFN     433
Prochlorococcus_phage_P-SSP7_gp15    ---------------------------G---EEFEGDIENFRDINLDE------TIKKVN     837
Escherichia_phage_BA14_gp15          ------------------------------------------------------------     759
Escherichia_phage_T7_gp15            ------------------------------------------------------------     747
Escherichia_phage_13a_gp15           ------------------------------------------------------------     747
Klebsiella_phage_K11_gp15            ------------------------------------------------------------     751
Yersenia_phage_phiYeO3-12_gp15       ------------------------------------------------------------     747
Escherichia_phage_K1E_gp35           ARKHVPPTRDWKIPFDQMHPAQQRGLMDLTYNLGKGGIQNSPRALAAFK--------AGK     882
Salmonella_phage_SP6_gp36            MKSHVPSTKDWAVPFDAMHPGVQRGLMDLSYNLGKDGIKNAPKAYAAFK--------AGK     877
Pseudomonas_phage_phiKMV_gp36        TDRALDQGVRLADELGVTNNASILGLAGMAFQMGEGRARQFRNTFQAIKDRNKEAFEAGV     857
Shigella_phage_Sf6_gp12              ------------------------------------------------------------     418
Salmonella_phage_P22_gp20            ------------------------------------------------------------     459
Salmonella_phage_HK620_gp20          ------------------------------------------------------------     436
Escherichia_phage_CUS-3_gp20-like    ------------------------------------------------------------     436

Salmonella_phage_Epsilon_15_gp12     TCEPFFEEFRL------------------YHRDENGKIVKTNDDVLDATRYGYMMRRFAR     475
Prochlorococcus_phage_P-SSP7_gp15    ------------------------------------------------------------     837
Escherichia_phage_BA14_gp15          ------------------------------------------------------------     759
Escherichia_phage_T7_gp15            ------------------------------------------------------------     747
Escherichia_phage_13a_gp15           ------------------------------------------------------------     747
Klebsiella_phage_K11_gp15            ------------------------------------------------------------     751
Yersenia_phage_phiYeO3-12_gp15       ------------------------------------------------------------     747
Escherichia_phage_K1E_gp35           LTEGFIEMLGTASSEGKRIPGLLKRRAEAYNMAAAG-GVPKITE-VETREDGSMWVKFGG     940
Salmonella_phage_SP6_gp36            FTDGFIEMLSTASTEGKRSSGLLVRRAEAYNLAQSGGSVPKISE-VETREDGSMYVKFSG     936
Pseudomonas_phage_phiKMV_gp36        RNSKWYTQ------TPDRAEAFIKRMAPHFDTP---------------------------     884
Shigella_phage_Sf6_gp12              -----------------------------PTTG---------------------------     422
Salmonella_phage_P22_gp20            -----------------------------YTSK---------------------------     463
Salmonella_phage_HK620_gp20          -----------------------------DSNK---------------------------     440
Escherichia_phage_CUS-3_gp20-like    -----------------------------DSNK---------------------------     440

Salmonella_phage_Epsilon_15_gp12     MMRDIRKPK--EKKIP---------A-P---IRPVRRGR---   499
Prochlorococcus_phage_P-SSP7_gp15    ------------------------------------------   837
Escherichia_phage_BA14_gp15          ------------------------------------------   759
Escherichia_phage_T7_gp15            ------------------------------------------   747
Escherichia_phage_13a_gp15           ------------------------------------------   747
Klebsiella_phage_K11_gp15            ------------------------------------------   751
Yersenia_phage_phiYeO3-12_gp15       ------------------------------------------   747
Escherichia_phage_K1E_gp35           PMPAGSVSAWTHKRIGADGWYQVYEAAPTKLAKDSKVGKVKL   982
Salmonella_phage_SP6_gp36            SMSEAFVSKSILGKIGKDGWMEVYPPKAGALASGTKVGRIKL   978
Pseudomonas_phage_phiKMV_gp36        ------------SQIG-VDWYSAATAE---------------   898
Shigella_phage_Sf6_gp12              ------------SNFS-SLWGD--------------------   431
Salmonella_phage_P22_gp20            ------------SGIK-FTVE---------------------   471
Salmonella_phage_HK620_gp20          ------------SGFS-SLWGD--------------------   449
Escherichia_phage_CUS-3_gp20-like    ------------SGFS-SLWGD--------------------   449
```

**Table S3.** Gp16-like sequence alignment.

```
Escherichia_phage_BA14_gp16           MDKYNPNEPHEYDALFQQAADTHGVSYGLLRKVGWVESRFKPTAQSPTGPRGVMQFTKAT     60
Escherichia_phage_T7_gp16             MDKYDKNVPSDYDGLFQKAADANGVSYDLLRKVAWTESRFVPTAKSKTGPLGMMQFTKAT     60
Escherichia_phage_13a_gp16            MDKYDKNVPSDYDGLFQKAADANGVSYDLLRKVAWTESRFVPTAKSKTGPLGMMQFTKAT     60
Klebsiella_phage_K11_gp16             -MSYDKSKPSDYDGIFQKAADSHGVSYDLLRKLSFNESSFNPKAVSKTGPKGIMQFTRNT     59
Yersenia_phage_phiYeO3-12_gp16        -MSYDKNKPSEFDGLFQKAADKHGVSYDLLRKLAFNESSFNPKAKSPTGPKGLMQFTKGT     59
Prochlorococcus_phage_P-SSP7_gp16     --------------------------------------------MSSDYQIDID---AQA     13
Escherichia_phage_K1E_gp36            ------------------------------------------------------------     0
Salmonella_phage_SP6_gp37             ------------------------------------------------------------     0
Pseudomonas_phage_phiKMV_gp37         -----------------------------------MAKQFKGRMT-PKYPLDQAQLDEAQ     24
Shigella_phage_Sf6_gp13               -----------------------------------MAKAWKDVIASPQYQ---ALAPEQK     22
Salmonella_phage_P22_gp16             ----------------------------------------------------------MK     2
Salmonella_phage_HK620_gp16           -----------------------------------MAKAWKDVVASPQYQ---ALTEEQK     22
Salmonella_phage_Epsilon_15_gp13      -----------------------------------MMK----VTANGK------------     9
Escherichia_phage_CUS-3_gp16-like     -----------------------------------MAKAWKDVIASPQYQ---ALTEEQK     22


Escherichia_phage_BA14_gp16           GQA-------YGLQN----DEDFTDPAKSIDAGARYLADLVK-KYDGDELKAALAYNQGE     108
Escherichia_phage_T7_gp16             AKA-------LGLRVTDGPDDDRLNPELAINAAAKQLAGLVG-KFDGDELKAALAYNQGE     112
Escherichia_phage_13a_gp16            AKA-------LGLRVTDGPDDDRLNPELAINAAAKHLAGLVG-KFDGDELKAALAYNQGE     112
Klebsiella_phage_K11_gp16             ARA-------MGLNVTDGDDDGRYNPELAIDAGAKLLASLVK-KYNGDELKAALAYNQGE     111
Yersenia_phage_phiYeO3-12_gp16        ATA-------LGLKVTDADDDDRYNPELAVDAAARHLSDLIR-KYDGDELKAALAYNQGE     111
Prochlorococcus_phage_P-SSP7_gp16     IQDSA-----LEFNEIYEENEK----------------------IEAQKR-----EQEL-     40
Escherichia_phage_K1E_gp36            ----------------MEQDIKTNWAG----YVQSTPEPF---SIEAAPVSAPTIRQRN-     36
Salmonella_phage_SP6_gp37             ----------------MADDISQSWVT----VSQRRLPPT---F-----------AQVA-     25
Pseudomonas_phage_phiKMV_gp37         VQGQLDAVPTVGFDALTGGEIGERNVA----AGQRANARELERIVADQEL--PA-LDRA-     76
Shigella_phage_Sf6_gp13               AQAQE-----QYFNEVVAPQAGENAEQ----AKQA--------FYAAYPL--PF-VQSV-     61
Salmonella_phage_P22_gp16             VTANG-----KTFNFPDG-TSTEDIGA----AVDE--------YFAGQAS--AA-ETQP-     40
Salmonella_phage_HK620_gp16           AQAQA-----QYFDEVVAPKAGDKWAE----ARDQ--------FYAA-------------     52
Salmonella_phage_Epsilon_15_gp13      ---TF-----TFPDGT----STEDIGT----AIDE--------YFAGQAV--QQ-Q-TV-     40
Escherichia_phage_CUS-3_gp16-like     AQAQA-----QYFDEVVAPKAGDKWAE----ARDQ--------FYAA-------------     52


Escherichia_phage_BA14_gp16           GRNGKPQLEAYDS------------GNFA----------SIG-DEGRNYLRSLLDVAKSP     145
Escherichia_phage_T7_gp16             GRLGNPQLEAYSK------------GDFA----------SIS-EEGRNYMRNLLDVAKSP     149
Escherichia_phage_13a_gp16            GRLGNQQLEAYSK------------GDFA----------SIS-EEGRNYMRNLLDVAKSP     149
Klebsiella_phage_K11_gp16             GPAGAPQLQAYDK------------GDFG----------SIS-EEGRNYMRKLLDVAKSP     148
Yersenia_phage_phiYeO3-12_gp16        GRNGAPQMQAYDK------------GDWA----------SIS-EEGRNYMRNLMDVANSP     148
Prochlorococcus_phage_P-SSP7_gp16     -LLQQQQEQAQA--EFDDPR----NKEGGGGLRGIS--KEIRSAIGGGLQDTASSIVTLP     91
Escherichia_phage_K1E_gp36            -ELQEQVLEAKADADILGAVGAAFQNEW--LAFGGKRWYDRATA--DFTPQPDFEIQPEQ     91
Salmonella_phage_SP6_gp37             -EAERKLEEQRAS---DKVMQTALESEW--ALYGGQRAIERHTT--EFAEQEGYTVPEST     77
Pseudomonas_phage_phiKMV_gp37         --------SAL-------WNQSTLVGRWGDAL---QLDADLA-ANSTGEVDPNFDAGTYG     117
Shigella_phage_Sf6_gp13               -ETQQPVAQQQ-------PQQSGFMSDL----------GEAVKETGRGLVQAGVNVANIP     103
Salmonella_phage_P22_gp16             --AEQQ-EEPQ-------QPEQSLMQRAGDLLTGGQSAGQIAEQAGRGLVNIPFDVLQGG     90
Salmonella_phage_HK620_gp16           ---YPP-HQQQ-------KEEPSLMQQAGDWLTGGQSAGQIAEQAGRGLVNIPFDVLQGG     101
Salmonella_phage_Epsilon_15_gp13      --NQAN-NAPT-------REEPSLMQQASDWLTGGQSAGQIAEQAGRGLVNIPFDVLQGG     90
Escherichia_phage_CUS-3_gp16-like     ---YPP-PQQQ-------KEEPSLMQQAGDWLTGGQSAGQIAEQAGRGLVNIPFDVLQGG     101
                                                                             .

Escherichia_phage_BA14_gp16           KSGDIESFGGITPKAKGIPFDAAMSGIGKKGKVTTELPESHE------MSFQ--------     191
Escherichia_phage_T7_gp16             MAGQLETFGGITPKGKGIPAEVGLAGIGHKQKVTQELPESTS------FDVK--------     195
Escherichia_phage_13a_gp16            MAGQLETFGGITPKGKGIPAEVGLAGIGHSQKVTQELPESTS------FDVK--------     195
Klebsiella_phage_K11_gp16             NSGALEAFGGITPKGKGIPAEDAFKGIAKAGKVGTELPESHG------FDIE--------     194
Yersenia_phage_phiYeO3-12_gp16        RKGDLEAFGGITPKAKGIPSGDAFAGIGKKQTVGTDLPESTG------FKVE--------     194
Prochlorococcus_phage_P-SSP7_gp16     ER-AIDMFSG-----------------EMVQE------QQTD------EGYGAEWDD-WF     120
Escherichia_phage_K1E_gp36            ----REALRF-------------KYGTDMMQTITEGVRSE----DELNFRIQNADEDL--     128
Salmonella_phage_SP6_gp37             ----KDELSK-------------IHGFEIAQDIVKDVKSP----EELQFRMSNAMADK--     114
Pseudomonas_phage_phiKMV_gp37         VQ-AL----------------------------QAAGIQPTDNYLQIMARAGNAKDAAYL     148
Shigella_phage_Sf6_gp13               AS-VADAVTSAAAWA--------GGKLGIGDGTYHPAPRVTTQGLEQDFGLQQG------     148
Salmonella_phage_P22_gp16             AS-LINAISQ----G--------LGGPKVLDDVYRPVDRPTDPYAQ--------------     123
Salmonella_phage_HK620_gp16           AS-LINAISQ----G--------LGGPKILDDVYRPVDRPTDPYAQ--------------     134
Salmonella_phage_Epsilon_15_gp13      AS-LINAISQ----G--------LGGPKVLDDVYRPVDRPTDPYAQ--------------     123
Escherichia_phage_CUS-3_gp16-like     AS-LINAISQ----G--------LGGPKVLDDVYRPAERPTDPYAQ--------------     134


Escherichia_phage_BA14_gp16           ----GKEQAAPNQPFGKDYWEAKGTTLDEANERSTFFGFGNAAEAELS------------     235
Escherichia_phage_T7_gp16             ----GIEQEATAKPFAKDFWETHGETLDEYNSRSTFFGFKNAAEAELS------------     239
Escherichia_phage_13a_gp16            ----GIEQEAPAKPFAKDFWETHGETLDEYNSRSTFFGFKNAAEAELS------------     239
Klebsiella_phage_K11_gp16             ----GVAQEAPNTPYAKDFWEKTGTTLDEYNSRSTFFGFGDAAEAQIQ------------     238
Yersenia_phage_phiYeO3-12_gp16        ----GKEQKAPNVPYAKDFWEKTGTTLDEFNARSTFFGIGDATSAELH------------     238
Prochlorococcus_phage_P-SSP7_gp16     VD---DANPIE----TKTWWGGALRSLVHFGSMAAAI-IPAAKVLGVTAASTAVGSLVRG     172
Escherichia_phage_K1E_gp36            ----ERNKRIAQAGWAGSVATIGAAVLDPVGWVASI---PTGGAAKVG----LVGRAVRG     177
Salmonella_phage_SP6_gp37             ----ERSEILARNGFTGFSAQLAAGIFDPVGWAASMVAAPVAGAVKVA----RVGRIIKT     166
Pseudomonas_phage_phiKMV_gp37         LSRIQRYEQDEQIVRDNPYWNFAAGMLDPAALAVDAVTFGAGRALRLG----RA-----G     199
Shigella_phage_Sf6_gp13               --ALT-----P-QTTEGRVFAEALPYLTPAGVERAAIQAP-TLAGRIA----QGATRLLA     195
Salmonella_phage_P22_gp16             ---------------AGE---SIGGYLIPG----------------AG----VA-----G     140
Salmonella_phage_HK620_gp16           ---------------AGE---TIGGYLLPIGTAAKAAGAPAKLAGDIG----SA-----G     167
Salmonella_phage_Epsilon_15_gp13      ---------------AGE---AIGGYLLPIGTAAKAAGAPAKLAGDIG----SA-----G     156
Escherichia_phage_CUS-3_gp16-like     ---------------AGE---TIGGYLLPIGTAAKAAGATAKLAGDIG----SA-----G     167
                                                                :

Escherichia_phage_BA14_gp16           ----NSTLGVAFRAGKRDNGFDVLTDVLQPTRFNSHIWTPEE---LNKIRTEVKNPAYMN     288
Escherichia_phage_T7_gp16             ----NSVAGMAFRAGRLDNGFDVFKDTITPTRWNSHIWTPEE---LEKIRTEVKNPAYIN     292
Escherichia_phage_13a_gp16            ----NSVAGMAFRAGRLDNGFDVFKDTITPTRWNSHIWTPEE---LEKIRTEVKNPAYIN     292
Klebsiella_phage_K11_gp16             ----NSTLGVAFRAARADDGYDVFKDTMTPTRWNSYVPSKED---LQKLRDSGLPPSYYG     291
```

```
Yersenia_phage_phiYeO3-12_gp16       ----NSVLGVAFRAARSDDGFDLFKDTITPTRWNSHTWTPEE---LERIRKEVKNPSYIN      291
Prochlorococcus_phage_P-SSP7_gp16    AG-VGAVSDVISKYSQEDNGLGILRDRFNFIDT--PISTKEHDHPAMKTLKNVVEGMGIG      229
Escherichia_phage_K1E_gp36           AIAAG-VSNAAI--------------------------------ESVLVQGDMTRDLDDI      204
Salmonella_phage_SP6_gp37            AAVAG-AENAAL--------------------------------EAILASGDYQKGADDV      193
Pseudomonas_phage_phiKMV_gp37        MAAAGGAGQVGY--------------------------------VAGLDAAGADVDAGTY      227
Shigella_phage_Sf6_gp13              ENAVGSLAA--N--------------------------------SAKDDAEALATDLGVG      221
Salmonella_phage_P22_gp16            NMAIGSVAEAAN--------------------------------QQGDFAGNVAKNAAVN      168
Salmonella_phage_HK620_gp16          NMIAGSLADAAN--------------------------------QDGDFAQNAAINGGIN      195
Salmonella_phage_Epsilon_15_gp13     NMIAGSLADAAN--------------------------------QEGDFAQNAAINGGIN      184
Escherichia_phage_CUS-3_gp16-like    NMIAGSLADAAN--------------------------------QEGDFAQNAAINGGIN      195
                                         .

Escherichia_phage_BA14_gp16          VVLGGSAENLDELIKMANENFEADAKAAEAGLGAKLSAGLIGAGVDPLTYVPIAGATAKG      348
Escherichia_phage_T7_gp16            VVTGGSPENLDDLIKLANENFENDSRAAEAGLGAKLSAGIIGAGVDPLSYVPMVGVTGKG      352
Escherichia_phage_13a_gp16           VVTGGSPENLDDLIKLANDNFENDSRAAEAGLGAKLSAGIIGAGVDPLSYVPMVGVTGKG      352
Klebsiella_phage_K11_gp16            VVTGGDGENWDALIKLAKDNFEADQRAAEAGTGAKLAAGIVGAGVDPLSYVPLVGVAGKG      351
Yersenia_phage_phiYeO3-12_gp16       VVTGGSPENLDALIKMANDNYEMDARSADAGVGAKLTAGIVGAGVDPLSYVPLVGVAGKG      351
Prochlorococcus_phage_P-SSP7_gp16    VVFDGLGIALGKGLRKTK---------------------INKAGQEV---------VEDG      259
Escherichia_phage_K1E_gp36           MVALGSGMAMGGVIGAVA---R--------------GRATKLSE-QG---------DDR-      236
Salmonella_phage_SP6_gp37            LAAAGFGMIMGGTIGAAT---R--------------ERIARKPGVQG---------VNDG      227
Pseudomonas_phage_phiKMV_gp37        IVAGALGAGVGALLGSGA------------------GRIAAEAPTQP--HVPEVSAPTVG      267
Shigella_phage_Sf6_gp13              VLAGGAINAAGRGLGAAYRGVR--------------GAIAPEAQQAI--RF--AEREGVP      263
Salmonella_phage_P22_gp16            LGAQGLLSGAAKLVGRGITAAR--------------GEIAPEARQLI--DT--AESMG--      208
Salmonella_phage_HK620_gp16          IGAQGVLSGVGRVIAPRVSQAL--------------GGAALNSANDV--SK--MAKSGTG      237
Salmonella_phage_Epsilon_15_gp13     IGAQGVLSGVGRVIAPRVSQAL--------------GGAALNSANDV--SR--MAKSGAG      226
Escherichia_phage_CUS-3_gp16-like    IGAQGVLSGVGRVIAPRVSQAL--------------GGAALNSANDV--SR--MAKSGAG      237
                                     :   .        :

Escherichia_phage_BA14_gp16          FKLV---------------------NKALVVGAQAGMLNV---------ASEGLRTSVAG      378
Escherichia_phage_T7_gp16            FKLI---------------------NKALVVGAESAALNV---------ASEGLRTSVAG      382
Escherichia_phage_13a_gp16           FKLI---------------------NKALVVGAESAALNI---------ASEGLRTSVAG      382
Klebsiella_phage_K11_gp16            LKVV---------------------NKALLVGAQAGALSV---------ASEGIRTSVAG      381
Yersenia_phage_phiYeO3-12_gp16       LKVI---------------------NKAFVVGTQSAGLAI---------ASEGIRTSIAG      381
Prochlorococcus_phage_P-SSP7_gp16    VSDAVNR-------ATAR---EANVNAQIDEKAALQAQSM--------------R--GQY      293
Escherichia_phage_K1E_gp36           -------------------------------------------------AASIVRSADAG      247
Salmonella_phage_SP6_gp37            AETVVDD------------------LDTVVKGADEFDAS----------AAKAVREAMEY      259
Pseudomonas_phage_phiKMV_gp37        LPEVAMT----AEEAAARGFKAGDVVDLLDEGTVLSRIS-------------A-RVEQA-      308
Shigella_phage_Sf6_gp13              L----------------------HTTDLLQPTSRVGKMAQTTAENIPLAGTSGMRATQ--      299
Salmonella_phage_P22_gp16            V--------------------KPMTSDMIKPGNAFTRSLMQGGEG-ALLGTGGKRAEQ--      245
Salmonella_phage_HK620_gp16          REIIARQSANVSDEIAKAADTAGIDINALTPGMRSGSRGLAQAEG-ILASKPGITQDAHT      296
Salmonella_phage_Epsilon_15_gp13     RQSIASQAANVSEDVAKAAESAGIDINALTPGMRSGSRGIAQAEG-ALASTPGIVQDAHQ      285
Escherichia_phage_CUS-3_gp16-like    RQSIASQAANVSEDVAKAAESAGIDINALTPGMRSGSRGIAQAEG-ALASTPGIVQDAHQ      296


Escherichia_phage_BA14_gp16          GDAHYAEA---------------------------------------------ALAGM--      391
Escherichia_phage_T7_gp16            GDADYAGA---------------------------------------------ALGGF--      395
Escherichia_phage_13a_gp16           GDADYAGA---------------------------------------------ALGGF--      395
Klebsiella_phage_K11_gp16            GEAHYADA---------------------------------------------ALGGL--      394
Yersenia_phage_phiYeO3-12_gp16       GEAHYADA---------------------------------------------ALGGL--      394
Prochlorococcus_phage_P-SSP7_gp16    G--GYKNKPISDPWQASP----------------NSTGK-AADVYYQKQRIDTDWGSQHG      334
Escherichia_phage_K1E_gp36           D--RYVR-----------------------------------------------------      252
Salmonella_phage_SP6_gp37            D--AYMAVRSYEPLRAKEVDMDVAILSHLDDLKANSSVRMSASEKGKLKEQIRQLETEAA      317
Pseudomonas_phage_phiKMV_gp37        ------EI--------PA-------IP----------------------RRDTAFGDELH      325
Shigella_phage_Sf6_gp13              -----QEARS------QL-------VQ----------------------RFADKFGEYDP      319
Salmonella_phage_P22_gp16            ----Y-AIRS------KL-------LG----------------------DYFDRVGGYNP      265
Salmonella_phage_HK620_gp16          K--AFSEIES------K--------FN----------------------SALDEFGAEAG      318
Salmonella_phage_Epsilon_15_gp13     A--AFNEISS------K--------LS----------------------RNLDEFGAASG      307
Escherichia_phage_CUS-3_gp16-like    A--AFNEISS------K--------LS----------------------RNLDEFGAASG      318


Escherichia_phage_BA14_gp16          -------------------LLAGGLTAVADGVAAG-----------------------LR      409
Escherichia_phage_T7_gp16            -------------------VFGAGMSAISDAVAAG-----------------------LK      413
Escherichia_phage_13a_gp16           -------------------LFGAGMSAISDAVAAG-----------------------LK      413
Klebsiella_phage_K11_gp16            -------------------LFGAGMSALSDAVAAG-----------------------IR      412
Yersenia_phage_phiYeO3-12_gp16       -------------------LFGAGMSAISDAIAAG-----------------------VR      412
Prochlorococcus_phage_P-SSP7_gp16    STDSPFTQRQIENLSESADIAEKEMVELMKPFMSDARIQAEIQGLKSGQSLADKFYDSIR      394
Escherichia_phage_K1E_gp36           ------------------------------------------------------------      252
Salmonella_phage_SP6_gp37            T------------------IKGKKVDAVAEAAAAKG------AP----KSAADRLDLDVK      349
Pseudomonas_phage_phiKMV_gp37        S------------------LSGRKLSEVVDHLKTHA------E-----------------      344
Shigella_phage_Sf6_gp13              A------------------V-------VIDSLKAKT------SG----IRRAAG------      338
Salmonella_phage_P22_gp16            D-------------------------DIVKSMTSTV------GG----RKNAAG------      284
Salmonella_phage_HK620_gp16          T------------------ASE-KSAAIKQRVLASI------DK----MKNSEK------      343
Salmonella_phage_Epsilon_15_gp13     T------------------ASE-KSAAIKQRILQNL------DQ----MKDAER------      332
Escherichia_phage_CUS-3_gp16-like    T------------------ASE-KSAAIKQRILQNL------DQ----MKDAER------      343


Escherichia_phage_BA14_gp16          KSGA-EQIENPFAAAQMRFEARETARNTGGYDASRM------------------------      444
Escherichia_phage_T7_gp16            RSKPEAEFDNEFIGPMMRLEARETARNANSADLSRM------------------------      449
Escherichia_phage_13a_gp16           RSKPEAEFDNEFIGPMMRMEARETARNANSADLSRM------------------------      449
Klebsiella_phage_K11_gp16            KARG-VDSVNEFAGPALRMEARETAINTGGHDTSTL------------------------      447
Yersenia_phage_phiYeO3-12_gp16       RSRG-TEVVNDFAPMAHRLEARETALNSGGEDLTRM------------------------      447
Prochlorococcus_phage_P-SSP7_gp16    RAHEVMNGRERLEDIDPDMFAAFDARSDTI---K------GKKVWQTADVLAADFVIGAL      445
Escherichia_phage_K1E_gp36           ------------------------------------------------------------      252
Salmonella_phage_SP6_gp37            KKALARRFDEPLADIQTRLDE----LNAKLARVENVGKSKEELK---------------R      390
Pseudomonas_phage_phiKMV_gp37        -------VPKPLQGIAAKV-------ADTLKTLEGLGQRTAFRVVQGGDTASS------A      384
```

```
Shigella_phage_Sf6_gp13                  --NRLEQVQNAMAGINIQPARAIQQIDTEISNLQKLGKVADNETISK-------------     383
Salmonella_phage_P22_gp16                --AVRDEIVNRMGSAPVGTTNSINAIDTNIARLEKLGTSADQRLLTA-------------     329
Salmonella_phage_HK620_gp16              --AAWDSVRSTMPDAKARMSNLNATIQGDI--------LAGMPLTPE-------------     380
Salmonella_phage_Epsilon_15_gp13         --AAWDDVRSTMPNQKARMLNGNAVIQAER--------SAGIPLTPE-------------     369
Escherichia_phage_CUS-3_gp16-like        --AAWDDVRSTMPNQKARMLNGNAVIQAER--------SAGIPLTPE-------------     380


Escherichia_phage_BA14_gp16              -----------------------------------------------------------P     445
Escherichia_phage_T7_gp16                -----------------------------------------------------------N     450
Escherichia_phage_13a_gp16               -----------------------------------------------------------N     450
Klebsiella_phage_K11_gp16                -----------------------------------------------------------P     448
Yersenia_phage_phiYeO3-12_gp16           -----------------------------------------------------------P     448
Prochlorococcus_phage_P-SSP7_gp16        FRKARDHGIAGRE-LFEIADLADIDGPAKALYDTLVGAVIQRKIASYTRGMELKNLDVRN     504
Escherichia_phage_K1E_gp36               ------------------------------------------------------------     252
Salmonella_phage_SP6_gp37                FS-----NLTREQQI----KELGLDAPARKV-EMTS--AVREALA----AI--RAEKKKT     432
Pseudomonas_phage_phiKMV_gp37            FLKPGTAGLHSTQGLDTLVQVRGSTAPGRVGTNPVT--VLHEAVHAATVGV--MNAALRN     440
Shigella_phage_Sf6_gp13                  --------L--QSYRDELVRNAGPDGPVNLDLKQLS--DLRSQFRMDVKGE--R---PVL     426
Salmonella_phage_P22_gp16                --------L-----KN----LKGELNSGNVDFDLLQ--QHRTAFRTNVQGD--A---MVF     365
Salmonella_phage_HK620_gp16              --------M--KQFAS----AYAKTGKNGITFDAMK--AWRSKLADAEQKY--I-----R     417
Salmonella_phage_Epsilon_15_gp13         --------M--KQFVQ----AN---NQGGVTFDGMK--AWRAKFADAEQKY--K-----R     403
Escherichia_phage_CUS-3_gp16-like        --------M--KQFVQ----AN---NQGGVTFDGMK--AWRAKFADAEQKY--K-----R     414


Escherichia_phage_BA14_gp16              PSEDRV-------------FSQHNGVEYAPLET----------EPGAVVLKDGSIISDTN     482
Escherichia_phage_T7_gp16                TENMKF-------------EGEHNGVPYEDLPT----------ERGAVVLHDGSVLSASN     487
Escherichia_phage_13a_gp16               TENMKF-------------EGEHNGVPYEDLPT----------ERGAVVLHDGSVLSASN     487
Klebsiella_phage_K11_gp16                PENFSF-------------EQDHRGVPFADHPT----------EEGAVVLANGSILSDTN     485
Yersenia_phage_phiYeO3-12_gp16           SENRVF-------------DREHAGVEYSPLET----------EPGAVVLPQGQILSDTN     485
Prochlorococcus_phage_P-SSP7_gp16        PANKQA------------------------------------------------------     510
Escherichia_phage_K1E_gp36               ------------------------AVADDSIGAMRVKGEE-------VLTEGAFDISSKG     281
Salmonella_phage_SP6_gp37                PTQTHA-------EAKAQAEEEVRQKRDDSIGAKRVEDSE--------IAGEQFDLSDSM     477
Pseudomonas_phage_phiKMV_gp37            PGAMSP-----KVAQAMQTLENVRGNVLNALKQDRAAGRQLSEFEETLLAGNSNTLANVK     495
Shigella_phage_Sf6_gp13                  PNRSDAAIQ--RVYKAM--TDDINGAIGQNLGNDTLR---------KYQQANAVYADEAA     473
Salmonella_phage_P22_gp16                PNQAKAATN--MVENAM--TRDLRNAVGKSLGPQDAA---------KYLKSNSDFANIYN     412
Salmonella_phage_HK620_gp16              SGEANTARRMAELRDAA--TEDMR-----------------------IMAQNGGFLDDWQ     452
Salmonella_phage_Epsilon_15_gp13         SGEANAARRAGEIRRAI--TDDMR-----------------------TMAENGGFLDDWQ     438
Escherichia_phage_CUS-3_gp16-like        SGEANAARRAGEIRRAI--TDDMR-----------------------TMAENGGFLDDWQ     449


Escherichia_phage_BA14_gp16              -----------LANPMTAKEFAEVDPERAAWGLPMRGL----------------------     509
Escherichia_phage_T7_gp16                -----------PINPKTLKEFSEVDPEKAARGIKLAGF----------------------     514
Escherichia_phage_13a_gp16               -----------PINPKTLKEFSEVDPEKAARGIKLAGF----------------------     514
Klebsiella_phage_K11_gp16                -----------PLNPRTQRDFAEIDPERAAPGIKLGGF----------------------     512
Yersenia_phage_phiYeO3-12_gp16           -----------PLNPQTLSEFEAVNPERAARGISLGGF----------------------     512
Prochlorococcus_phage_P-SSP7_gp16        ------------------------------------------------------------     510
Escherichia_phage_K1E_gp36               EDLLKTLQRE--------GNAIDMTPRRWAGTMSALGT----------------------     311
Salmonella_phage_SP6_gp37                EDLMDDLARE--------AYQSEVRPVNLKG-LGSVSS----------------------     506
Pseudomonas_phage_phiKMV_gp37            ELVAWGLTDTRFQRTLNRLRYSDGGPGLWSR--FVEGIRSLLGLRSDADTALSRVLAASE     553
Shigella_phage_Sf6_gp13                  K--LKN---TRLKNVL--MKG-DLTPEVVNNMLFSKN------------------KSEIK     507
Salmonella_phage_P22_gp16                K--VLN---KRISNTLNKARS-EYTPELINTVVFSR-----------------K-PSDIK     448
Salmonella_phage_HK620_gp16              K--ANDLSKARFT-AQEQAEA-AFGRDLATDQLVTNGSKALQGSAK------SG-TGQFH     501
Salmonella_phage_Epsilon_15_gp13         K--ANDLSKARLS-AQESAES-VFGRDLATDALITNGVKSLQSSSA---KGLNG-PAGFH     490
Escherichia_phage_CUS-3_gp16-like        K--ANDLSKARLS-AQESAES-VFGRDLATDALITNGVKSLQSSSA---KGLNG-PAGFH     501


Escherichia_phage_BA14_gp16              ----------------SEIGLRTL------RSEHAEIRGLAKD-LVRSPTGMESGSHGKF     546
Escherichia_phage_T7_gp16                ----------------TEIGLKTL------GSDDADIRRVAID-LVRSPTGMQSGASGKF     551
Escherichia_phage_13a_gp16               ----------------TDIGLKTL------GSDDADIRRVAID-LVRSPTGMQSGASGKF     551
Klebsiella_phage_K11_gp16                ----------------TEIGLKTL------GSKDAGVRAIAQD-LVRSPTGMQSGSSGKF     549
Yersenia_phage_phiYeO3-12_gp16           ----------------TEIGLKTL------RSENPTVRSIASD-LVRSPTGMESGTSGKF     549
Prochlorococcus_phage_P-SSP7_gp16        --------------LKDGINAE------------------IE------KTK--LAYQVAF     530
Escherichia_phage_K1E_gp36               VVHSSQDA------SVRGLGARLFES----------------------PQG--LGMQKA-     340
Salmonella_phage_SP6_gp37                VILNSKNP------VFRGLGLRLLEN----------------------AQG--GAYQGK-     535
Pseudomonas_phage_phiKMV_gp37            TIMDAMPGYTKAQAKWANKGAPV--------TEEASLETIVQSTRERAREG--AGFVNRF     603
Shigella_phage_Sf6_gp13                  TLYNSVGRVGRAQMRNGIIG------------------KAM-------EKS--GGSPDQF     540
Salmonella_phage_P22_gp16                RIWSSLDNKGK-------------DAMR-----AAYISKIAEK---TGD------SPAKF     481
Salmonella_phage_HK620_gp16              KIISALPESERAPAIASILQDTVSQGVRGGKSEGAGIKHIATI---LTPQN--VKAISRY     556
Salmonella_phage_Epsilon_15_gp13         SMIRALPESERVPAISSMLQDAISHGVRGGKSDAAGIHHIAGI---LTPQN--VKAISRY     545
Escherichia_phage_CUS-3_gp16-like        SMIRALPESERVPAISSMLQDAISHGVRGGKADAAGINHIAEI---LTPQN--VKAISRY     556


Escherichia_phage_BA14_gp16              GAT-----ASDIKER--------LHSNNQRTYNDLYGAM-KEAMADPEW-----------     581
Escherichia_phage_T7_gp16                GAT-----ASDIHER--------LHGTDQRTYNDLYKAM-SDAMKDPEF-----------     586
Escherichia_phage_13a_gp16               GAT-----ASDIHER--------LHGTDQRTYNDLYKAM-SDAMKDPEF-----------     586
Klebsiella_phage_K11_gp16                GAT-----ASDIHER--------LHATDQRMYNQLYDAV-DRAMKDPEF-----------     584
Yersenia_phage_phiYeO3-12_gp16           GAT-----ASDIKER--------LHANNQRTYNQLYDAV-RTAMKDPEF-----------     584
Prochlorococcus_phage_P-SSP7_gp16        KYAGDNQDDSLFRAYYEA-----VSMSNDIHNFDDFDAFIKRKLKGGELNGKVKTGVLIK     585
Escherichia_phage_K1E_gp36               --S-----ASLMQNTNLNR----LKSADMNRFNDGFDLWLKEN-----------------     372
Salmonella_phage_SP6_gp37                --T-----ASILSNVYGNL----IRFAEKNRYNDGFSQFIKDN-----------------     567
Pseudomonas_phage_phiKMV_gp37            FSE-----ADLLAQPGEGARRLLGRLIDDPVRRDGFSTND--------------------     638
Shigella_phage_Sf6_gp13                  LRQ-----LNILQNQTGI----------------TFKG----------------------     557
Salmonella_phage_P22_gp16                ITE-----VNKLKAQSGGE-----------IYNTIFSG----------------------     503
Salmonella_phage_HK620_gp16              SPE-----LGRIASSYGEL----ARAATKPLRYVEQTG----------------------     585
Salmonella_phage_Epsilon_15_gp13         SSE-----LGRIADAYGTL----ARAAVKPQQYIERTG----------------------     574
Escherichia_phage_CUS-3_gp16-like        SSE-----LGRIADAYGTL----ARAAVKPQQYIERTG----------------------     585
```

```
                                                 . :

Escherichia_phage_BA14_gp16            ----------------SVGM-----FKQGAQGARQEIYRRVAIAIER----------PEL        610
Escherichia_phage_T7_gp16              ----------------STGG-----AKMSREETRYTIYRRAALAIER----------PEL        615
Escherichia_phage_13a_gp16             ----------------STGG-----AKMSREEARYTIYRRAALAIER----------PEL        615
Klebsiella_phage_K11_gp16              ----------------SVGE-----QKMSRRAIRQEVYKRASLAIER----------PEL        613
Yersenia_phage_phiYeO3-12_gp16         ----------------STGG-----ATMSRKEIRQEIYKRAALAIER----------PEL        613
Prochlorococcus_phage_P-SSP7_gp16      ELQGVMINSVLSGPKTSVRAIMGTGTATFLRPFSQVIG-ATL------------------        626
Escherichia_phage_K1E_gp36             ----------------NINPVAGHTNSHYVQQYNEKVWEAVRIGMDE-------------        403
Salmonella_phage_SP6_gp37              ----------------NLRAVDY-LNPAVTRDFNNQIYTAIVKGIPD-------------        597
Pseudomonas_phage_phiKMV_gp37          ------------------------NAASYLRRYRNEF-EGYVKSYDEMMAKAMAEQGVGL        673
Shigella_phage_Sf6_gp13                ------------------------QDAAYLKGLKN-----YLQSTQ--------------        574
Salmonella_phage_P22_gp16              ------------------------RHMKELDALHD-----VL--------RQTA------        520
Salmonella_phage_HK620_gp16            ------------------------RSMPAISTLEK-----GLHPVLESVLSGAF------        610
Salmonella_phage_Epsilon_15_gp13       ------------------------RTANVLRDLDA-----GLSNVTSTVLNAIA------        599
Escherichia_phage_CUS-3_gp16-like      ------------------------RTANVLRDLDA-----GLSNVTSTVLNAIA------        610


Escherichia_phage_BA14_gp16            QANLTKAE--RKVMDIMKEHFDLKREMMENPSM---FGNK--------------------        645
Escherichia_phage_T7_gp16              QKALTPSE--RIVMDIIKRHFDTKRELMENPAI---FGNTK-------------------        651
Escherichia_phage_13a_gp16             QKALTPSE--RIVMDIIKRHFDTKRELMENPAI---FGNTK-------------------        651
Klebsiella_phage_K11_gp16              QADLTKGE--REVMDLLKEHFDTKRELMEQPGI---FGNAN-------------------        649
Yersenia_phage_phiYeO3-12_gp16         QANLTKGE--RNVMNILKQHFDLKREIMENPSI---FGNTK-------------------        649
Prochlorococcus_phage_P-SSP7_gp16      TGDKTTQRAALAAMSGMM---------ESIPEAWKVFST----------------KLNSY        661
Escherichia_phage_K1E_gp36             --------------------------------------------ATPKSIRMAAEGQQAM        419
Salmonella_phage_SP6_gp37              --------------------------------------------DTPRGVKLAAEGIADK        613
Pseudomonas_phage_phiKMV_gp37          TARALNSRRAMAVRDQLNE--QVTRELLRRDREWTAYGSVRVDPNLPPTIKALADRSDEI        731
Shigella_phage_Sf6_gp13                -----------------------------------------------------QAAKAAV        581
Salmonella_phage_P22_gp16              -----------------------------------------------------RSDSANV        527
Salmonella_phage_HK620_gp16            ------------------------------------------------------PTAGAI        616
Salmonella_phage_Epsilon_15_gp13       -----------------------------------------------------NSTSGAI        606
Escherichia_phage_CUS-3_gp16-like      -----------------------------------------------------NSTSGAI        617


Escherichia_phage_BA14_gp16            -----------ANSIFPNSRHKGTYVPHVYSREAKQLYSQALGGSDGLQEAIA--ASW-M        691
Escherichia_phage_T7_gp16              -----------AVSIFPESRHKGTYVPHVYDRHAKALMIQR-YGAEGLQEGIA--RSW-M        696
Escherichia_phage_13a_gp16             -----------AVSIFPESRHKGTYVPHVYDRHAKALMIQR-YGVEGLQEGIA--RSW-M        696
Klebsiella_phage_K11_gp16              -----------AVSIFPGSRHKGTYVPNVYDRGAKELMMQKLGGPEGLQQAIA--QSW-L        695
Yersenia_phage_phiYeO3-12_gp16         -----------AVSIFPGSRHKGTYVPNVYDRAIKIEMVKR-YDNDGLQRAIA--ESW-L        694
Prochlorococcus_phage_P-SSP7_gp16      W----SGDISTIKTRFNQVTKGDEQWAMLGDWIENSGKAN--AGDKAAYYMANQARALND        715
Escherichia_phage_K1E_gp36             YREALALRQRSGEAGFEKVKADDKYMPDIFDSMKARRQFG-MHDKEDIIELFS--RAYQN        476
Salmonella_phage_SP6_gp37              LAKSLEIRKAAGEKGFEDVKSARDYIPVIYDGIKVTEAVNRLGSSEAVIALLS--KGYQT        671
Pseudomonas_phage_phiKMV_gp37          HGLMGQRAREAGVRGFEDFAPRPGYFHRSWNWSKMA------QMDEAAPGLAR--RAISE        783
Shigella_phage_Sf6_gp13                TTPTGQQ-TIPFII---------GYGTAMN---PAT------TGAAVSYGLLT--RAYES        620
Salmonella_phage_P22_gp16              VTQTGQALANPVRL---------GAAIPTL---GKS------LAAEAGYGLAM--RVYES        567
Salmonella_phage_HK620_gp16            AGFSGGGVIGAI------------------------------VGGAAG--------GAID        638
Salmonella_phage_Epsilon_15_gp13       VGGAGGGIAG----------------------------------AAAG--------ALVG        624
Escherichia_phage_CUS-3_gp16-like      VGGAGGGIAG----------------------------------AAAG--------ALVG        635


Escherichia_phage_BA14_gp16            TSYRSRPEVKARVDEHLAETLGID-VKAVTEEMVMKHASDKAYGIAKTDQFNSSSVIDDN        750
Escherichia_phage_T7_gp16              NSYVSRPEVKARVDEMLKELHGV---KEVTPEMVEKYAMDKAYGISHSDQFTNSSIIEEN        753
Escherichia_phage_13a_gp16             NSYVSRPDVKARVDEMLKEVHGV---KEVTPEMVEKYAMDKAYGISHSDQFTNSSIIEEN        753
Klebsiella_phage_K11_gp16              TSYRVRPEVKARVDEYLMELNGYKSVDQVTPEVVQKHAMDKAYGISHTEDFTASSVIDDN        755
Yersenia_phage_phiYeO3-12_gp16         TSYRARPEVKARVDEYLMELNGLKSVHEVTPEMVQKHAMDKAYGISHTDQFSASSVIEDN        754
Prochlorococcus_phage_P-SSP7_gp16      NKFLT-----------------------------------------------YST----K        724
Escherichia_phage_K1E_gp36             ----GARKIPKEVADEIARAQVN---RVVDATLTGRMSFEKAMSGQTKAEYEAIM----R        525
Salmonella_phage_SP6_gp37              ----GKYKMGKKAADALAKVQYI---RASDSTLSSRVAFDRVVSQQQQAQLIEDL----K        720
Pseudomonas_phage_phiKMV_gp37          AVFRGIPGLDRADADTIAQAIVQ---RARDRATGIRSEFMGAMGVADTAFIRQAL----E        836
Shigella_phage_Sf6_gp13                EPFRN-------------------------------------------------------        625
Salmonella_phage_P22_gp16              KPIRN-------------------------------------------------------        572
Salmonella_phage_HK620_gp16            AIAKG-------------------------------------------------------        643
Salmonella_phage_Epsilon_15_gp13       AGLKG-------------------------------------------------------        629
Escherichia_phage_CUS-3_gp16-like      AGLKG-------------------------------------------------------        640


Escherichia_phage_BA14_gp16            IEGLVGIENNSFLEARNLFD----------SDMPVT-----LPNGQQFSVNDLRDFDMKH        795
Escherichia_phage_T7_gp16              IEGLVGIENNSFLEARNLFD----------SDLSIT-----MPDGQQFSVNDLRDFDMFR        798
Escherichia_phage_13a_gp16             IEGLVGIENNSFLEARNLFD----------SDLSIT-----MPDGQQFSVNDLRDFDMFR        798
Klebsiella_phage_K11_gp16              ITGLVGIENNSFLEARNMFD----------SDLPVT-----LPDGSTFSVNDLRDFDMAR        800
Yersenia_phage_phiYeO3-12_gp16         IEGLVGIENNNFLEARNMFD----------SDMAVT-----LPDGNTFSVNDLRTYDMAE        799
Prochlorococcus_phage_P-SSP7_gp16      IMAATDDTFGFLLARSRAKE--------K--AMRLAM-------------EQLNKGNITE        761
Escherichia_phage_K1E_gp36             KAGFSDEEIEKMVEALDNKETKDNISNRAKMSLGLDVTQ----EYNGIRMRDFMNTNVEE        581
Salmonella_phage_SP6_gp37              RAGVPDNIIDNFIEGTELQEMAESVSNRAKASMGINTQA----EYGGMKVQDLLNTNVGE        776
Pseudomonas_phage_phiKMV_gp37          EANVSQAKFDSIMAKIEQKQSDQGTVKYGKGRLSLDMTAEINHNGTVYRVQDLIDRDLDR        896
Shigella_phage_Sf6_gp13                ------------------------------------------------------------        625
Salmonella_phage_P22_gp16              ------------------------------------------------------------        572
Salmonella_phage_HK620_gp16            ------------------------------------------------------------        643
Salmonella_phage_Epsilon_15_gp13       ------------------------------------------------------------        629
Escherichia_phage_CUS-3_gp16-like      ------------------------------------------------------------        640


Escherichia_phage_BA14_gp16            VMPAYDRRVDGDIAIMGGTGKTTAELKDAIMALDKKSEGKG-------------------        836
Escherichia_phage_T7_gp16              IMPAYDRRVNGDIAIMGSTGKTTKELKDEILALKAKAEGDG-------------------        839
Escherichia_phage_13a_gp16             IMPAYDRRVNGDIAIMGSTGKTTKELKDEILALKAKAEGDG-------------------        839
```

```
Klebsiella_phage_K11_gp16              IIPAYDRRVNGDISIMGGSGKTTQQLKDEIMALDKRAERKG-------------------     841
Yersenia_phage_phiYeO3-12_gp16         ILPAYDRRVDGDVAIMGGSGKTTKDLKDEIMALDKQSEGNG-------------------     840
Prochlorococcus_phage_P-SSP7_gp16      ITPDLLKNAQDRFY--AQITDADGNITEAATLFAKKEATLTTD-----------------     802
Escherichia_phage_K1E_gp36             LTDNYMKEAAGGAALARQGFSTYQAALNAIDLVERNARNAAKDTK-AHAQFEAESAKIRQ     640
Salmonella_phage_SP6_gp37              LAENYGKEAAGGAALAAMGFPTRQSVLNAIDAAERAGRNMAGADAKAIK-----------     825
Pseudomonas_phage_phiKMV_gp37          LMENYAGSMSGRSALARAGMPGDSEIEAFIREYQREAAHLGTD-----------------     939
Shigella_phage_Sf6_gp13                AMLRMANTPRGSTAFEKAMQQSQKAINALTQGAKS-------------------------     660
Salmonella_phage_P22_gp16              MLLRLANTKPGTPAYERALNQAATAVRP--------------------------------     600
Salmonella_phage_HK620_gp16            SIAKLSATRSGRYAIEKAVQEATKAVKVGA---SD-------------------------     675
Salmonella_phage_Epsilon_15_gp13       AVSKIATTRSGRYAIEKAVQEATKAVRAGG---SK-------------------------     661
Escherichia_phage_CUS-3_gp16-like      AVSKIATTRSGRYAIEKAVQEATKAVRAGG---SK-------------------------     672
                                                 .

Escherichia_phage_BA14_gp16            ------------------------------TMKGEVEALKDTVKILTGRARRNHD-TVGD     865
Escherichia_phage_T7_gp16              ------------------------------KKTGEVHALMDTVKILTGRARRNQD-TVWE     868
Escherichia_phage_13a_gp16             ------------------------------KKTGEVHALMDTVKILTGRARRNQD-TVWE     868
Klebsiella_phage_K11_gp16              ------------------------------QLKGEVEALKDTVKILTGRARRNND-TAFE     870
Yersenia_phage_phiYeO3-12_gp16         ------------------------------TLKGEVEALKDTVKILTGRARRNPE-GAWG     869
Prochlorococcus_phage_P-SSP7_gp16      -----------------------------------LTGFSKGLNDVFEAAPWAKPFFLFA     827
Escherichia_phage_K1E_gp36             SEPDYKKAQEKIEELKKRLKLKEKDEAAGLAIDEEIRQMEGRLRLIMGKSIDADPQALST     700
Salmonella_phage_SP6_gp37              ------------------------------QLRAESEMLRDSVKLIYGNTIDANPNAGIV     855
Pseudomonas_phage_phiKMV_gp37          ------------------------------KVQELTGQLRGVFGDFTGNVPREH---QLG     966
Shigella_phage_Sf6_gp13                ------------------------------------DALSE-------------------     665
Salmonella_phage_P22_gp16              ---------------------------------------------LLANEATRQ------     609
Salmonella_phage_HK620_gp16            ------------------------------------GSLAAAERRFMANKAAVK---AI-     695
Salmonella_phage_Epsilon_15_gp13       ------------------------------------EALAAAERRFMANKAAVK---AI-     681
Escherichia_phage_CUS-3_gp16-like      ------------------------------------EALAAAERRFMANKAAVK---AI-     692

Escherichia_phage_BA14_gp16            TMF------------------R--------ALSDMSFFTKNAYMGLQNLTEI-SGLLAKG     898
Escherichia_phage_T7_gp16              TSL------------------R--------AINDLGFFAKNAYMGAQNITEI-AGMIVTG     901
Escherichia_phage_13a_gp16             TSL------------------R--------SINDLGFFAKNAYMGAQNITEI-AGMIVTG     901
Klebsiella_phage_K11_gp16              TAM------------------R--------TLNDLAFFAKNFYMGPQNLTEI-AGMLAKG     903
Yersenia_phage_phiYeO3-12_gp16         TAL------------------R--------SVNDLTFFAKNAYMGAQNVTEI-AGMLAKG     902
Prochlorococcus_phage_P-SSP7_gp16      RTGVNGLSLTAKHTPGFNFLVKEWNEIAFADPSNLSGLQRYGIETAEDLANAKALQVGRL     887
Escherichia_phage_K1E_gp36             KMLR--------------------------RGRDITGVLRLGQMGFAQLGEL-ANFMGEF     733
Salmonella_phage_SP6_gp37              RGTR--------------------------RVREITGLLRLGQMGFAQVPEL-ARAITKM     888
Pseudomonas_phage_phiKMV_gp37          PVAQ--------------------------RASGLTSATMLGFSGVYQLAEL-ATMAHRQ     999
Shigella_phage_Sf6_gp13                ------------------------------------------------------------     665
Salmonella_phage_P22_gp16              ------------------------------------------------------------     609
Salmonella_phage_HK620_gp16            -------------------------------------RDALGNEEFQRL--------SRA     710
Salmonella_phage_Epsilon_15_gp13       -------------------------------------RDAVGNEEFNRL--------ARA     696
Escherichia_phage_CUS-3_gp16-like      -------------------------------------RDAVGNEEFNRL--------ARA     707

Escherichia_phage_BA14_gp16            NTRAMLHGIPALRD---LAF---------RNKPVSGKELKELHSMVFGKEFDDLIRPARQ     946
Escherichia_phage_T7_gp16              NVRALGHGIPILRD---TLY---------KSKPVSAKELKELHASLFGKEVDQLIRPKRA     949
Escherichia_phage_13a_gp16             NVRALSHGIPILRD---TLY---------KSKPVSAKELKELHASLFGKEVDQLIRPKRA     949
Klebsiella_phage_K11_gp16              NVKAMLHGIPTLRD---LAT---------RTSPVSGSELRELHGALFGKELDQLIRPGRE     951
Yersenia_phage_phiYeO3-12_gp16         NVSAITHGIPMIND---WVN---------RGKPLRASEIKELHGMVFGKELDQLIRPGRE     950
Prochlorococcus_phage_P-SSP7_gp16      AIGSSVISMAALHFMNGGLTGNG-PADRQKRQAWI------DAGYK-PRT----I-----     930
Escherichia_phage_K1E_gp36             GIAATTIALGKQFRFTSKALR---SGDGFFRDKNLA-EVERMVGY-IGED----N-----     779
Salmonella_phage_SP6_gp37              GVGTVLKSIPATKFLRSRAGRKGGTAQGELLEPELR-EMEELIGY-IGED----N-----     937
Pseudomonas_phage_phiKMV_gp37          GVFNVMKAMLNS-----RMGD---FVGAMRRNPDLADEMQTVLGLNLAND----I-----     1042
Shigella_phage_Sf6_gp13                ------------------------------------------------------------     665
Salmonella_phage_P22_gp16              ------------------------------------------------------------     609
Salmonella_phage_HK620_gp16            GIV---------------------------------------------------------     713
Salmonella_phage_Epsilon_15_gp13       GIV---------------------------------------------------------     699
Escherichia_phage_CUS-3_gp16-like      GIV---------------------------------------------------------     710

Escherichia_phage_BA14_gp16            DIIQRLRESTDTPDLAAKVVGTIK-HTTQELAARSPFTKFLNG---TSNYILDMARQGVM     1002
Escherichia_phage_T7_gp16              DIVQRLREATDTGPAVANIVGTLK-YSTQELAARSPWTKLLNG---TTNYLLDAARQGML     1005
Escherichia_phage_13a_gp16             DIVQRLREATDTGPAVANIVGTLK-YSTQELAARSPWTKLLNG---TTNYLLDAARQGML     1005
Klebsiella_phage_K11_gp16              DIVQRIREASDTSGAMASVIGTIK-FGTQELSARSPWTKMLNG---TANYILDTARQGVL     1007
Yersenia_phage_phiYeO3-12_gp16         DHVRRLRESTDTSAAVANVVGTIR-FGTQELAARSPWTMLLNG---TSNYILDAARQGVL     1006
Prochlorococcus_phage_P-SSP7_gp16      -TIGGVQVSYDSFEPFNLI-----------------------------------------     948
Escherichia_phage_K1E_gp36             -WLTTKGARPDEFGDVTTVKGMMA-HFDQSMNSIRRAQTNLSLFRMAQGSLERMTNRQIA     837
Salmonella_phage_SP6_gp37              -WLSGWNVRHDEFGETADNMGRLSAIIDNGLAMGSRINTWLSGFKAIQGGSEKIVARSIN     996
Pseudomonas_phage_phiKMV_gp37          -RMKPWKRQFDTFLASQDT------FMDRFLHAGKQAVPVLNGMKFIHNWQSRMNANLTL     1095
Shigella_phage_Sf6_gp13                ------------------------------------------------------------     665
Salmonella_phage_P22_gp16              ------------------------------------------------------------     609
Salmonella_phage_HK620_gp16            --------------------------------------ASLSGMAQE-------------     722
Salmonella_phage_Epsilon_15_gp13       --------------------------------------ASLSGIAQE-------------     708
Escherichia_phage_CUS-3_gp16-like      --------------------------------------ASLSGMAQE-------------     719

Escherichia_phage_BA14_gp16            GDVVTHAITGKGANKWIKKDMLKSASISKEQWEGIQNLIRENVTQGADGKYTFKDKRKLA     1062
Escherichia_phage_T7_gp16              GDVISATLTGKT-TRWEKEGFLRGASVTPEQMAGIKSLIKEHMVRGEDGKFTVKDKQAFS     1064
Escherichia_phage_13a_gp16             GDVISATLTGKT-TRWEKEGFLRGASVTPEQMAGIKSLIKEHMVRGEDGKFTVKDKQAFS     1064
Klebsiella_phage_K11_gp16              GDVAGAALGGKG-SKFGKENFLKAASISPEQWKGIKQLFVDHATRDANGQFTIKDKKAFS     1066
Yersenia_phage_phiYeO3-12_gp16         GDVAGAALAGKA-SKFGKANYLKSASISPEQWNGIKQLFRDYATRGEDGKFTIRDKQAFA     1065
Prochlorococcus_phage_P-SSP7_gp16      -------------------------------------------------------LSTIA     953
Escherichia_phage_K1E_gp36             LSFIDHLEGKKIIPQ------------------------KKLEELGLTQEFMTNLQKHYD     873
Salmonella_phage_SP6_gp37              KRLKQHLMGERELPK------------------------RDLEEVGLDEATMKRLKRHFD     1032
```

```
Pseudomonas_phage_phiKMV_gp37          NKVARAAQG-DEAAL------------------------RVLQQYGKDVDWTPV----LA          1126
Shigella_phage_Sf6_gp13                ------------------------------------------------------------          665
Salmonella_phage_P22_gp16              ------------------------------------------------------------          609
Salmonella_phage_HK620_gp16            ------------------------------------------------------------          722
Salmonella_phage_Epsilon_15_gp13       ------------------------------------------------------------          708
Escherichia_phage_CUS-3_gp16-like      ------------------------------------------------------------          719

Escherichia_phage_BA14_gp16            NDP----------R--AMDLWRLADK-------------VADETMLRPHKVSLQDSHAFG          1097
Escherichia_phage_T7_gp16              MDP----------R--AMDLWRLADK-------------VADEAMLRPHKVSLQDSHAFG          1099
Escherichia_phage_13a_gp16             MDP----------R--AMDLWRLADK-------------VADEAMLRPHKVSLQDSHAFG          1099
Klebsiella_phage_K11_gp16              QDP----------R--AMDLWRLADK-------------VADETMLRPHKVSQQDSKAYG          1101
Yersenia_phage_phiYeO3-12_gp16         NDP----------R--SMDLWRLADK-------------VADETILRPHKVSSQDAKAFG          1100
Prochlorococcus_phage_P-SSP7_gp16      DVGDYSQLMGEEWTEDQFQKLA-----LVVAQGISSKSYMAGIQQFVDLFGGQAGSWERI          1008
Escherichia_phage_K1E_gp36             ANSK-------GSGLLGFDTMPYAMG-ETLANAIRR---KSGL-IIQRNFIGDEGIWMNK          921
Salmonella_phage_SP6_gp37              ENPMYADYNGEKVRMMNFDAMEPDLR-EIVGVAVRR---MSGR-LIQRNFIGDEGIWMNK          1087
Pseudomonas_phage_phiKMV_gp37          RVRGYVTYRGRNAQSMNWGAWSQADVNTVMNTALRI---MDDS-LL-YGRVGQNSGFARS          1181
Shigella_phage_Sf6_gp13                ------------------------------------------------------------          665
Salmonella_phage_P22_gp16              ------------------------------------------------------------          609
Salmonella_phage_HK620_gp16            ------------------------------------------------------------          722
Salmonella_phage_Epsilon_15_gp13       ------------------------------------------------------------          708
Escherichia_phage_CUS-3_gp16-like      ------------------------------------------------------------          719

Escherichia_phage_BA14_gp16            AAAKLVLQFKSFVIK--------SMNAKFIRSGHEAMKNHRAMDMA--------------          1135
Escherichia_phage_T7_gp16              ALGKMVMQFKSFTIK--------SLNSKFLRTFYDGYKNNRAIDAA--------------          1137
Escherichia_phage_13a_gp16             ALGKMVMQFKSFTIK--------SLNSKFLRTFYDGYKNNRAIDAA--------------          1137
Klebsiella_phage_K11_gp16              AGVKMAMQFKNFTIK--------SLNAKFIRSFYEGYKNNRAIDMA--------------          1139
Yersenia_phage_phiYeO3-12_gp16         AGVKMVMQFKNFVIK--------SLNSRFVRSFYEATKNNRALDQA--------------          1138
Prochlorococcus_phage_P-SSP7_gp16      ISGLINNQIPLSSLRNELGKVFNPHMKELNSGIIESIRNRNLISEGLAINELPTKYDMLN          1068
Escherichia_phage_K1E_gp36             ALGKTFAQLKSFSLV--------SGEK----QFGRGIRH-----------------DKIG          952
Salmonella_phage_SP6_gp37              WWGKALTQFKSFSIV--------SIEK----QLIHDLRG-----------------DKIQ          1118
Pseudomonas_phage_phiKMV_gp37          PVGQILGQFRSFVAF--------AHNK----LLRGTYEN----------------SGVLG          1213
Shigella_phage_Sf6_gp13                ------------------------------------------------------------          665
Salmonella_phage_P22_gp16              ------------------------------------------------------------          609
Salmonella_phage_HK620_gp16            ------------------------------------------------------------          722
Salmonella_phage_Epsilon_15_gp13       ------------------------------------------------------------          708
Escherichia_phage_CUS-3_gp16-like      ------------------------------------------------------------          719

Escherichia_phage_BA14_gp16            LTYAISGGIAGSYYVAQAHLKAAGLPKEQQKDYLKKALDPKMIAYAAASRSSHLGSPLSI          1195
Escherichia_phage_T7_gp16              LSIITSMGLAGGFYAMAAHVKAYALPKEKRKEYLERALDPTMIAHAALSRSSQLGAPLAM          1197
Escherichia_phage_13a_gp16             MSIVTSMGLAGGFYAMAVHVKAYALPKEKRKEYLERALDPTMIAHAALSRSSHLGAPLAM          1197
Klebsiella_phage_K11_gp16              LTHVLSLGIAGTYFAMQAHVKAYGLQESQRKDYLKKALNPTMLGYAALTRSSHTGAPLSI          1199
Yersenia_phage_phiYeO3-12_gp16         LTHIISLGLAGGYYVGQAHLKAASLQEHKRKEYLKNALDPKMIAHASISRSSHLGSPLSI          1198
Prochlorococcus_phage_P-SSP7_gp16      GRPIKDWDFPTRMFNMFSPFYVNLDQSEGRKLLFNSGYDLRMSTYSSPDGID-LSDNARL          1127
Escherichia_phage_K1E_gp36             LAKKTAYGFALGSIVYAAKAYVNSIGREDQDEYLEEKLSPKG--------LA--------          996
Salmonella_phage_SP6_gp37              AAQIMAWSSLLGFASYATQMQMQAIGREDRDKFLREKFDTQN--------IA--------          1162
Pseudomonas_phage_phiKMV_gp37          VASLLAFQYPLTALMMGAKAAI------------NGKFDTSD------EGIR--------          1247
Shigella_phage_Sf6_gp13                ------------------------------------------------------------          665
Salmonella_phage_P22_gp16              ------------------------------------------------------------          609
Salmonella_phage_HK620_gp16            ------------------------------------------------------------          722
Salmonella_phage_Epsilon_15_gp13       ------------------------------------------------------------          708
Escherichia_phage_CUS-3_gp16-like      ------------------------------------------------------------          719

Escherichia_phage_BA14_gp16            ANFAMGAAGYDQG-----------------------------------------------          1208
Escherichia_phage_T7_gp16              VDLVGGVLGFESS-----------------------------------------------          1210
Escherichia_phage_13a_gp16             ADLVGGALGFESS-----------------------------------------------          1210
Klebsiella_phage_K11_gp16              VSMMAGAAGFQDA-----------------------------------------------          1212
Yersenia_phage_phiYeO3-12_gp16         YDMFAGMVGSDTY-----------------------------------------------          1211
Prochlorococcus_phage_P-SSP7_gp16      RSLYMKAIGDQNLEAKLNKLAKNPKVIASIEKMQADLRAGKKEINPRTAYVHNKMIHTLF          1187
Escherichia_phage_K1E_gp36             ----FGAMG--------------------------------------------MMSTTAV          1008
Salmonella_phage_SP6_gp37              ----MGVFN--------------------------------------------KLPQVAG          1174
Pseudomonas_phage_phiKMV_gp37          ----KMAID--------------------------------------------GIGYTAG          1259
Shigella_phage_Sf6_gp13                ------------------------------------------------------------          665
Salmonella_phage_P22_gp16              ------------------------------------------------------------          609
Salmonella_phage_HK620_gp16            ------------------------------------------------------------          722
Salmonella_phage_Epsilon_15_gp13       ------------------------------------------------------------          708
Escherichia_phage_CUS-3_gp16-like      ------------------------------------------------------------          719

Escherichia_phage_BA14_gp16            ------LMVRSTILPKGDDK-RDRNKAVTSRDMGDSIMGAIGEQVPALGFAGATLAAGRN          1261
Escherichia_phage_T7_gp16              ------KMARSTILPKDTVKERDPNKPYTSREVMGAMGSNLLEQMPSAGFVANVGATLMN          1264
Escherichia_phage_13a_gp16             ------KMARSTILPKDTTKERDPNKPYTSREVMGAMGSNLLEQMPSAGFVANVGATLMN          1264
Klebsiella_phage_K11_gp16              ------NMLRSTILPKEEQF-QKKDGASKGRAESSNLAGNLGSQVPALGYVGNVIATAKN          1265
Yersenia_phage_phiYeO3-12_gp16         ------KYTRSTVLPKESEK-RDPNKAMTGRQVASTIAGAVGEQVPGLGFVGSVGATAIN          1264
Prochlorococcus_phage_P-SSP7_gp16      MEARKIAWAKVRNDPEALQL-------YAEDKRINIQN-------------ETS------          1221
Escherichia_phage_K1E_gp36             FSLGGDFLGGLGVLPSELVQ-------S--RYEAGFQTKGLIDQIPLVGVGQDA------          1053
Salmonella_phage_SP6_gp37              FGLAGDTFATFGLMPDSMMQ-------A--PGRMGFRQQGFGDLVAGAGVISDA------          1219
Pseudomonas_phage_phiKMV_gp37          LGFTADMWGVVT---GH--------------SRMSA---------PVFGLAEHS------          1287
Shigella_phage_Sf6_gp13                ------------------------------------------------------------          665
Salmonella_phage_P22_gp16              ------------------------------------------------------------          609
Salmonella_phage_HK620_gp16            ------------------------------------------------------------          722
Salmonella_phage_Epsilon_15_gp13       ------------------------------------------------------------          708
```

```
Escherichia_phage_CUS-3_gp16-like      ------------------------------------------------------------        719


Escherichia_phage_BA14_gp16            AYGVLTAPNKPTER----EMMTGLMNAHREMIPNDP---ISQQMLIKFYEANGVHIKADK        1314
Escherichia_phage_T7_gp16              AAGVVNSPNKATEQ----DFMTGLMNSTKELVPNDP---LTQQLVLKIYEANGVNLRERR        1317
Escherichia_phage_13a_gp16             AAGVVNSPNKATEQ----DFMTGLMNSTKELVPNDP---LTQQLVLKIYEANGVNLRERR        1317
Klebsiella_phage_K11_gp16              AYGVATAPNKPTER----DYMTGLMNSTKELVPNDP---LTQQLIMKIYEANGVTIKQQP        1318
Yersenia_phage_phiYeO3-12_gp16         AASLLSSPNKATAL----EFRTGLFNTSRELIPNDP---LSQQLIMKIYEANGIRIKETP        1317
Prochlorococcus_phage_P-SSP7_gp16      ----LNTTRNYTNQNAES-------NPSNLLLPYR-------------------------        1245
Escherichia_phage_K1E_gp36             -YRLADSITKYAEGDTE-G--VDVARRALRLVPLTNVIGIQNALRYG------LDELED-        1102
Salmonella_phage_SP6_gp37              -VNLSQALVKYANGDDD-VSTRQLVDKVRRLVPLANTIGVGQMTKAS------VDLLED-        1270
Pseudomonas_phage_phiKMV_gp37          -NEVFRGVKDLVTGDDPAAATGDIVSGAAGALPFVNVFPATKLLLES------IKGE---        1337
Shigella_phage_Sf6_gp13                ------------------------------------------------------------        665
Salmonella_phage_P22_gp16              ------------------------------------------------------------        609
Salmonella_phage_HK620_gp16            ------------------------------------------------------------        722
Salmonella_phage_Epsilon_15_gp13       ------------------------------------------------------------        708
Escherichia_phage_CUS-3_gp16-like      ------------------------------------------------------------        719


Escherichia_phage_BA14_gp16            K--      1315
Escherichia_phage_T7_gp16              K--      1318
Escherichia_phage_13a_gp16             K--      1318
Klebsiella_phage_K11_gp16              KPN      1321
Yersenia_phage_phiYeO3-12_gp16         KKQ      1320
Prochlorococcus_phage_P-SSP7_gp16      ---      1245
Escherichia_phage_K1E_gp36             ---      1102
Salmonella_phage_SP6_gp37              ---      1270
Pseudomonas_phage_phiKMV_gp37          ---      1337
Shigella_phage_Sf6_gp13                ---      665
Salmonella_phage_P22_gp16              ---      609
Salmonella_phage_HK620_gp16            ---      722
Salmonella_phage_Epsilon_15_gp13       ---      708
Escherichia_phage_CUS-3_gp16-like      ---      719
```
